# Oxidation of Protein Kinase A Regulatory Subunit PKARIα Protects Against Myocardial Ischemia-Reperfusion Injury by Inhibiting Lysosomal-Triggered Calcium Release

**DOI:** 10.1161/CIRCULATIONAHA.120.046761

**Published:** 2020-11-13

**Authors:** Jillian N. Simon, Besarte Vrellaku, Stefania Monterisi, Sandy M. Chu, Nadiia Rawlings, Oliver Lomas, Gerard A. Marchal, Dominic Waithe, Fahima Syeda, Parag R. Gajendragadkar, Raja Jayaram, Rana Sayeed, Keith M. Channon, Larissa Fabritz, Pawel Swietach, Manuela Zaccolo, Philip Eaton, Barbara Casadei

**Affiliations:** 1 Division of Cardiovascular Medicine, Radcliffe Department of Medicine (J.N.S., B.V., S.M.C., N.R., O.L., G.A.M., P.R.G., R.J., K.M.C., B.C.), University of Oxford, United Kingdom.; 2 Department of Physiology, Anatomy and Genetics (S.M., P.S., M.Z.), University of Oxford, United Kingdom.; 3 Wolfson Imaging Centre, Weatherall Institute of Molecular Medicine (D.W.), University of Oxford, United Kingdom.; 4 Institute of Cardiovascular Sciences, University of Birmingham, United Kingdom (F.S., L.F.).; 5 Cardiothoracic Surgery, Oxford Heart Centre, Oxford University Hospitals National Health Service Foundation Trust, United Kingdom (R.S.).; 6 Department of Cardiology, University Hospitals Birmingham, United Kingdom (L.F.).; 7 William Harvey Research Institute, Barts and the London School of Medicine and Dentistry, Queen Mary University of London, Charterhouse Square, United Kingdom (P.E.).

**Keywords:** calcium signaling, lysosome, protein kinase A phosphorylation, redox, reperfusion injury

## Abstract

**Background:**

Kinase oxidation is a critical signaling mechanism through which changes in the intracellular redox state alter cardiac function. In the myocardium, PKARIα (type-1 protein kinase A) can be reversibly oxidized, forming interprotein disulfide bonds in the holoenzyme complex. However, the effect of PKARIα disulfide formation on downstream signaling in the heart, particularly under states of oxidative stress such as ischemia and reperfusion (I/R), remains unexplored.

**Methods:**

Atrial tissue obtained from patients before and after cardiopulmonary bypass and reperfusion and left ventricular (LV) tissue from mice subjected to I/R or sham surgery were used to assess PKARIα disulfide formation by immunoblot. To determine the effect of disulfide formation on PKARIα catalytic activity and subcellular localization, live-cell fluorescence imaging and stimulated emission depletion super-resolution microscopy were performed in *prkar1* knock-out mouse embryonic fibroblasts, neonatal myocytes, or adult LV myocytes isolated from “redox dead” (Cys17Ser) PKARIα knock-in mice and their wild-type littermates. Comparison of intracellular calcium dynamics between genotypes was assessed in fura2-loaded LV myocytes, whereas I/R-injury was assessed ex vivo.

**Results:**

In both humans and mice, myocardial PKARIα disulfide formation was found to be significantly increased (2-fold in humans, *P*=0.023; 2.4-fold in mice, *P*<0.001) in response to I/R in vivo. In mouse LV cardiomyocytes, disulfide-containing PKARIα was not found to impact catalytic activity, but instead led to enhanced AKAP (A-kinase anchoring protein) binding with preferential localization of the holoenzyme to the lysosome. Redox-dependent regulation of lysosomal two-pore channels by PKARIα was sufficient to prevent global calcium release from the sarcoplasmic reticulum in LV myocytes, without affecting intrinsic ryanodine receptor leak or phosphorylation. Absence of I/R-induced PKARIα disulfide formation in “redox dead” knock-in mouse hearts resulted in larger infarcts (2-fold, *P*<0.001) and a concomitant reduction in LV contractile recovery (1.6-fold, *P*<0.001), which was prevented by administering the lysosomal two-pore channel inhibitor Ned-19 at the time of reperfusion.

**Conclusions:**

Disulfide modification targets PKARIα to the lysosome, where it acts as a gatekeeper for two-pore channel–mediated triggering of global calcium release. In the postischemic heart, this regulatory mechanism is critical for protection from extensive injury and offers a novel target for the design of cardioprotective therapeutics.

## Abstract 2

Supplemental Digital Content is available in the text.

**Clinical Perspective**

**What Is New?**

- We offer the first evidence that ischemia/reperfusion injury, in humans and in mice, induces PKARIα (regulatory subunit Iα-containing protein kinase A) oxidation and disulfide formation.
- Disulfide formation enhances PKARIα intracellular anchoring and promotes compartmentation of the holoenzyme complex to the lysosome, where it acts as a negative regulator of two-pore channel–dependent calcium release.
- Using genetic loss of PKARIα disulfide formation, we demonstrate that this newly identified regulatory mechanism serves as a crucial, adaptive response to myocardial ischemia/reperfusion injury by inhibiting excess calcium release and limiting infarct size.

**What Are the Clinical Implications?**

- Inhibition of lysosomal two-pore channel–dependent calcium release by oxidized PKARIα prevents myocardial cell death in response to ischemia/reperfusion, revealing a previously unrecognized mechanism of cardioprotection that could be exploited for therapeutic intervention.

**Editorial, see p 466**

Oxidative stress plays a pivotal role in the pathogenesis of ischemia-reperfusion (I/R) injury, with early bursts of reactive oxygen species (ROS) initiating a cascade of deleterious cellular processes that promote cell death and cardiac dysfunction.^1,2^ Paradoxically, prevention of ROS generation by inhibiting specific oxidase systems exacerbates I/R injury,^3,4^ suggesting that some degree of ROS formation is necessary for cardioprotection.^2,5^ Evidence that ROS underpin the effects of preconditioning or some cardioprotective compounds^6–8^ supports this conclusion, as does the general failure of antioxidants to reduce reperfusion injury after coronary angioplasty^9^ or improve clinical outcomes in patients with acute myocardial infarction or heart failure.^10^ Although it is known that ROS signaling is mediated largely through covalent modification of specific cysteine thiols within redox-sensitive proteins,^11^ the exact mechanisms through which they exert their cardioprotective actions remain unclear.

Protein kinase A (PKA) is 1 of the master regulatory molecules in the heart. Under physiological conditions, PKA contributes to the cardiac response to catecholamine stimulation through catalyzed phosphorylation of proteins involved in excitation-contraction coupling, metabolism, and cardiomyocyte hypertrophy.^12,13^ In disease states, however, persistent activation of PKA signaling, or altered expression of PKA isotypes, has been linked to maladaptive remodeling, pathological hypertrophy, and the progression to heart failure,^14^ making pharmacological targeting of PKA an attractive therapy for the treatment of cardiac disease.

The ability for PKA to regulate a multitude of cellular processes occurs through differential expression and localization of 2 distinct isotypes (type-1 and type-2) composed of 2 catalytic (PKA_cat_) and 2 regulatory subunits (RIα or RIβ and RII, respectively).^15^ Although all PKA isotypes depend on cAMP binding for activation, recent work has shown that PKARIα (type-1 PKA) possesses 2 cysteine residues within the RIα subunits that are sensitive to ROS-mediated oxidation.^16,17^ Studies in isolated hearts and cardiomyocytes, using exogenous oxidants, have shown that oxidation of these cysteines leads to formation of an interprotein disulfide bond within the RIα subunit,^17^ which may enhance the holoenzyme’s catalytic activity, independent of cAMP, or promote PKARIα subcellular targeting.^16,18^ Beyond this, however, little else is known about the endogenous triggers of PKARIα disulfide formation in the myocardium or how PKARIα oxidation affects cardiac function.

Here, we provide the first evidence for endogenous induction of PKARIα disulfide formation in the heart, occurring after I/R in both humans and mice. Using high spatial and temporal resolution imaging modalities, in conjunction with a “redox dead” PKARIα knock-in (KI) mouse model,^19^ we demonstrate that disulfide modification targets PKARIα to the lysosome, where it acts as a gatekeeper for two-pore channel (TPC)–mediated Ca^2+^ release and prevents inappropriate triggering of Ca^2+^ release from the sarcoplasmic reticulum (SR). In the postischemic heart, we find that inhibition of lysosomal Ca^2+^ release by oxidized PKARIα is crucial for limiting infarct size and preserving cardiac function during reperfusion, offering a novel target for the design of cardioprotective therapeutics.

## Methods

Supporting data and methods can be found in Methods in the Data Supplement and will be made available, on reasonable request, by contacting the corresponding author.

### Human Samples

Biopsies of the right atrial appendage were obtained before and after cardiopulmonary bypass and reperfusion in patients undergoing on-pump coronary artery bypass surgery at the John Radcliffe Hospital (Oxford, United Kingdom). The study was approved by the Research Ethics Committee (reference no. 07/Q1607/38), and all patients gave written, informed consent.

### Animals

“Redox dead” PKARIα KI mice (C57BL/6 background), in which the nucleotides encoding for cysteine at position 17 were mutated to nucleotides encoding for serine (Cys17Ser), were generated as previously described.^19^ Only male mice were used for assessment of infarct size. For all other studies, KI mice (12–18 weeks old) of both sexes were compared with their wild-type (WT) littermates. All experiments involving animals were carried out in accordance with the United Kingdom Home Office Guidance on the Operation of Animals (Scientific Procedures) Act of 1986 and approved by the University of Oxford Ethics Committee.

### Statistical Analysis

All experimentation and data analysis, apart from immunoblots, were conducted blinded to genotype and intervention. Data were checked for normality of distribution before statistical analysis using a Shapiro-Wilk normality test. Comparisons between data were performed using either a Student *t* test or ANOVA with Bonferroni correction (normally distributed) or using the Mann-Whitney test or Kruskal-Wallis test (nonnormally distributed). For Ca^2+^ handling data in cardiomyocytes, analyses were carried out in RStudio using a hierarchical statistical method,^20^ taking into consideration clustering of single cells per animal and correcting for this in the statistical analysis. The incidence of spontaneous Ca^2+^ release events was compared using a Fisher exact test. A *P* value <0.05 was considered statistically significant.

## Results

### Myocardial I/R Promotes PKARIα Disulfide Formation

**Figure 1.**
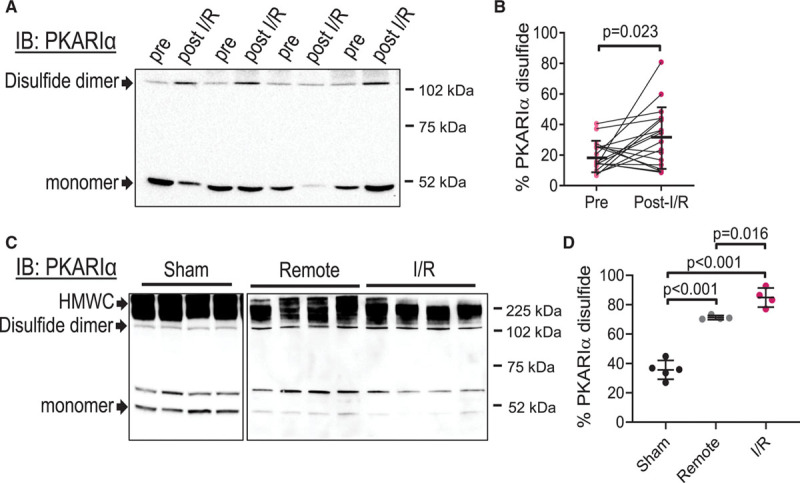
**Myocardial I/R induces PKARIα oxidation in humans and mice.** **A**, Immunoblot detection of PKARIα monomers and disulfide dimers, observed under nonreducing conditions, in atrial biopsies taken just before cardiopulmonary bypass (pre) and again immediately after reperfusion (post-I/R) in patients undergoing on-pump cardiac surgery. **B**, Quantification of the percentage of PKARIα that exists as a disulfide dimer (calculated by the density of PKARIα dimer/(the density of dimer + monomer) and expressed as a percentage of total PKARIα) shows significant elevations in the disulfide state following I/R in humans. Mean±SD; paired Student *t* test. n=18. **C**, Immunoblot detection of PKARIα monomers, disulfide dimers, and high molecular weight complexes (HMWC), observed under nonreducing conditions, in mouse left ventricular samples obtained remotely or downstream of a transient coronary artery ligation (for 45 minutes occlusion and 30 minutes reperfusion [I/R]) or after sham surgery. The presence of a second band above the monomer was found to be a nonspecific reaction (Figure IC in the Data Supplement). **D**, Quantification of the percentage of PKARIα disulfide dimers (calculated as in **B**) shows higher levels of oxidation in the I/R and remote tissue compared with sham-operated hearts. Data are mean±SD; 1-way ANOVA; n=5 for sham, n=4 for remote and I/R. IB indicates immunoblot; and PKARIα, regulatory subunit Iα-containing protein kinase A.

Although PKARIα disulfide formation is known to occur in the heart in response to exogenous oxidant treatment,^16–18^ no evidence for endogenous induction of PKARIα oxidation and disulfide formation has been reported. We therefore aimed to determine whether, in humans and mice, disease states associated with increased ROS production would promote PKARIα disulfide bond formation. Atrial tissue biopsies taken from patients undergoing on-pump cardiac surgery showed a minimal degree of PKARIα disulfide formation before cardiopulmonary bypass; however, in samples acquired from the same patient minutes after cardioplegia and reperfusion, the PKARIα disulfide state was found to be significantly increased (Figure 1A and 1B).

Left ventricular (LV) tissue obtained from mice undergoing transient coronary artery ligation in vivo also displayed markedly enhanced PKARIα disulfide formation compared with sham-operated mice, with increased PKARIα oxidation seen in both the I/R and the remote region of the LV (Figure 1C and 1D). Control experiments in human and mouse tissue showed a reduction of high molecular weight bands when samples were treated with reducing agents (Figure IA and IB in the Data Supplement), confirming that the higher molecular weight bands were disulfide dimerized PKARIα. In mice, a second band just above the putative RIα monomer was also found after reduction. Further experiments revealed this to be a nonspecific band no longer present when PKARIα was purified using cAMP-affinity capture (Figure IC in the Data Supplement). Unlike oxidative modifications that lead to protein degradation,^21^ PKARIα oxidation was not associated with loss of total PKARIα protein levels (as observed in Figure IA and IB in the Data Supplement), suggesting that this modification is regulatory in nature and likely has a functional role during I/R-injury.

### RIα Disulfide Formation Enhances PKA Intracellular Anchoring Through A-Kinase Anchoring Protein Binding Without Affecting Catalytic Activity

For several kinases, regulatory oxidation of cysteine thiols increases their catalytic activity.^21^ To test whether this was the case for PKARIα, we used real-time monitoring of PKA catalytic activity by the genetically encoded AKAR3ev fluorescence resonance energy transfer (FRET) biosensor, which we expressed in cultured adult LV cardiomyocytes isolated from PKARIα “redox dead” KI mice or their WT littermates. Before use of the KI mouse model for mechanistic studies, detailed cardiac characterization was undertaken to rule out gross structural cardiac remodeling (Figure IIA through IID in the Data Supplement), neurohumoral abnormalities (Figure IIE through IIH in the Data Supplement), or alterations in baseline cardiac function (Table I in the Data Supplement).

**Figure 2.**
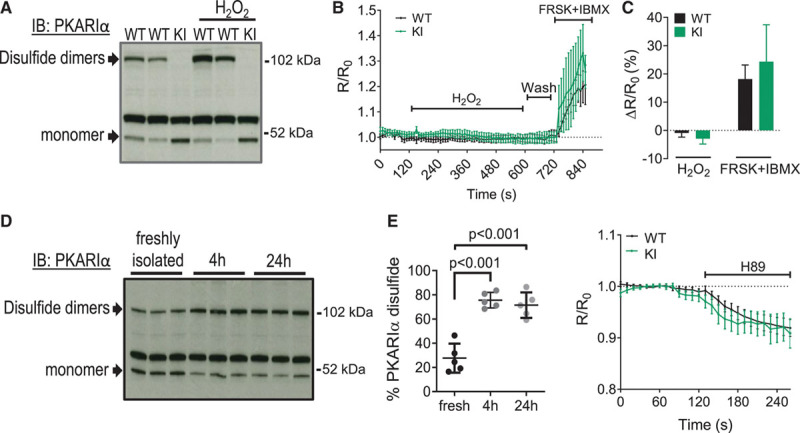
**PKARIα disulfide bond formation does not affect global catalytic activity.** **A**, Immunoblot detection of PKARIα monomers and disulfide dimers, observed under nonreducing conditions, in WT and KI left ventricular cardiomyocytes treated with vehicle or H_2_O_2_ (250 µmol/L, 10 minutes). H_2_O_2_ induced formation of PKARIα disulfides in WT, but not in KI, cardiomyocytes (isolated from n=11 mice for WT and n=6 for KI). **B**, Dynamic measurement of protein kinase A (PKA) activity in cultured adult mouse cardiomyocytes using the cytosolic fluorescence resonance energy transfer (FRET) AKAR3ev biosensor (expressed as a normalized yellow fluorescent protein/cyan fluorescent protein FRET ratio, R/R_0_) following addition of H_2_O_2_ (250 µmol/L), during H_2_O_2_ wash off, and in response to maximal stimulation of cAMP production by forskolin (FRSK; 25 µmol/L) plus isobutylmethylxanthine (IBMX; 100 µmol/L). Mean±SEM; 2-way ANOVA with repeated measures; *P*<0.01 for time-effect, *P* value not significant for genotype or interaction. n=6 or 7 cardiomyocytes per group from 4 mice per genotype. **C**, Quantification of the maximal FRET ratio in WT and KI cardiomyocytes during H_2_O_2_ and FRSK + IBMX treatment. **D**, Induction of PKARIα disulfide dimer formation, assessed under nonreducing conditions, following 4 hours and 24 hours of culturing. Cardiomyocytes from the same isolation were used for statistical comparison at each time. All data points shown with mean±SEM indicated; 1-way ANOVA with Bonferroni correction; n=5. **E**, Assessment of basal PKA activity using the AKAR3ev FRET biosensor and the PKA inhibitor H89 (30 µmol/L) in cardiomyocytes cultured for 24 hours. Data are mean±SEM; 2-way ANOVA with repeated measures; *P*<0.01 for time-effect, *P* value not significant for genotype or interaction. n=6 or 7 cardiomyocytes per group from 4 mice per genotype. IB indicates immunoblot; KI, knock-in; PKARIα, regulatory subunit Iα-containing protein kinase A; and WT, wild-type.

As demonstrated in Figure 2A, genetic substitution of a serine for 1 of the critical, disulfide-forming cysteines in the RIα subunit (Cys17Ser)^19^ prevented KI mice from forming PKARIα disulfide bonds, either under basal conditions or in response to H_2_O_2_ treatment. By contrast, freshly isolated WT cardiomyocytes showed a significant proportion of RIα in the disulfide state under basal conditions (52.6±3.6%), which was further increased by treatment with H_2_O_2_ (83.0±2.1%). Despite this marked difference in PKARIα disulfide state between WT and KI cardiomyocytes, we found no change in the normalized FRET ratio over the course of the 8-minute H_2_O_2_ incubation (Figure 2B and 2C). Addition of saturating doses of forskolin and 3-isobutyl-1-methylxanthine at the end of each protocol confirmed that the sensor responded appropriately to a rise in intracellular cAMP and further indicated that no oxidant-induced potentiation of forskolin/3-isobutyl-1-methylxanthine activation occurred (Figure 2C).

Cardiomyocyte culture itself, which was necessary to allow adenoviral gene transduction of the FRET sensor, was associated with a significant increase in the proportion of RIα disulfide formation (up to 72.1±5.8% after 24 hours in culture; Figure 2D). PKARIα was found to be highly oxidized following culture or storage of cells under all ex vivo conditions assessed (Table II in the Data Supplement). As the near-complete induction of disulfide bond formation by culturing could have accounted for the failure of PKARIα activity to increase in response to H_2_O_2_, we also tested whether, in WT cardiomyocytes, PKARIα exhibited greater intrinsic catalytic compared with KI, as evaluated using the H89-inhibitable fraction. As shown in Figure 2E, the FRET response to H89 did not differ between WT and KI cardiomyocytes, consistent with the overall conclusion that disulfide formation has no direct effect on PKARIα catalytic activity.

Given that disulfide bonds form within the A-Kinase Anchoring Protein (AKAP)–binding domain of the RIα subunit,^22^ we asked whether PKARIα intracellular anchoring was impacted by the oxidation state. To assess this, we conducted fluorescence recovery after photobleaching experiments—which offer the robust capability of measuring protein diffusion and mobility in live cells^23^—in PKARIα knock-out (*prkar1a*^−/−^) mouse embryonic fibroblasts expressing green fluorescent protein–tagged WT or mutant RIα proteins. The use of *prkar1a*^−/−^ mouse embryonic fibroblasts allowed us to monitor changes in intracellular anchoring of green fluorescent protein–tagged PKARIα in the absence of endogenous PKARIα, which might compete for available AKAP-binding sites. Nonreduced immunoblotting confirmed the presence of disulfide bond formation in cells expressing PKARIα(WT) or in cells expressing PKARIα(H24A), a mutation known to substantially reduce PKARIα AKAP-binding affinity without affecting disulfide formation,^22^ but not in PKARIα(C17S)-expressing cells (Figure IIIA in the Data Supplement).

**Figure 3.**
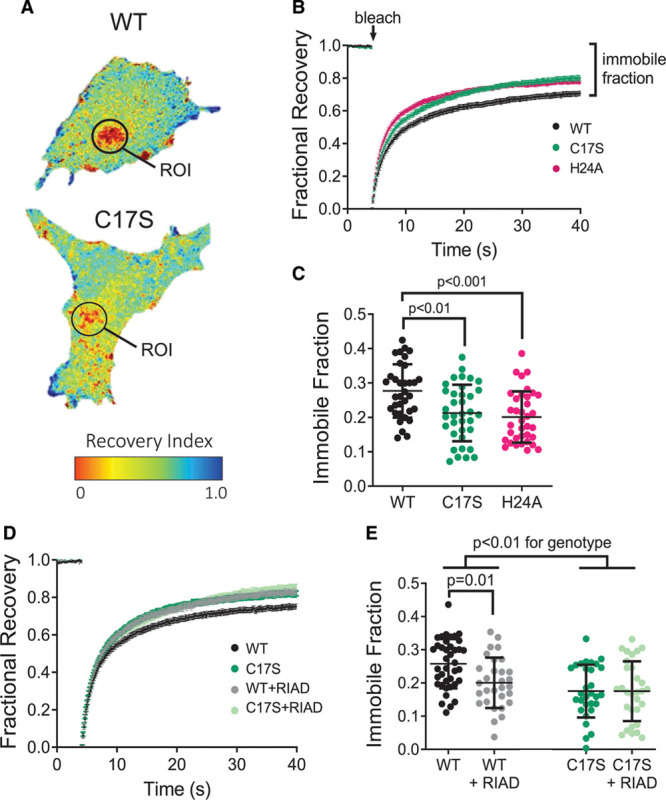
**PKARIα disulfide bond formation enhances intracellular anchoring.** **A**, Expression of the photobleached recovery index, which provides a ratio of GFP- PKARIα fluorescence before bleaching and at the end of the recovery period (per pixel) in *prkar1a*^−/−^ mouse embryonic fibroblast (MEF) cells expressing WT or C17S PKARIα. Fluorescence recovery after photobleaching region of interest (ROI) as indicated. **B**, Fluorescence recovery dynamics, with time of bleach and the fraction of fluorescence that does not recover (eg, immobile fraction) indicated. **C**, Quantification of the immobile fraction within the ROI (shown with mean±SD) for *prkar1a*^−/−^ MEF cells expressing WT (black), C17S (green), and H24A (pink) GFP-tagged PKARIα. One-way ANOVA; n=30 to 39 cells per group, from 3 independent passages. **D**, Fluorescence recovery dynamics and (**E**) quantification of the immobile fraction (shown as mean±SD) from *prkar1a*^−/−^ MEF cells expressing WT (black/gray) or C17S (green/light green) in the absence or presence of cotransfection with the RIα anchoring disruptor (RIAD) peptide. Two-way ANOVA with Bonferroni posttest; n=30 to 39 cells per group, from 3 independent passages. GFP indicates green fluorescent protein; PKARIα, regulatory subunit Iα-containing protein kinase A; and WT, wild-type.

Compared with PKARIα(WT), PKARIα(C17S) showed a higher degree of green fluorescent protein–PKARIα diffusive exchange within the photobleached region of interest, as indicated by the higher recovery index (Figure 3A). This difference was reflected quantitatively as a reduction in the immobile (ie, anchored) fraction of PKARIα in C17S-expressing cells compared with WT (Figure 3B and 3C), indicating that, in the absence of disulfide formation, less PKARIα is restricted to intracellular compartments. The relative reduction in the immobile fraction for PKARIα(C17S)-expressing cells was equivalent to that found in PKARIα(H24A)-expressing cells (Figure 3C). Diffusion rate constants for the mobile fraction of PKARIα were also calculated from the fluorescence recovery after photobleaching curves but were found not to differ between mutant and WT PKARIα (Figure IIIB in the Data Supplement).

To test whether the reduction in immobile PKARIα(C17S) was a result of a loss of disulfide-dependent anchoring to endogenous AKAPs, we repeated the fluorescence recovery after photobleaching experiments in cells expressing each of the constructs (WT or C17S) in combination with the RIα anchoring disruptor (RIAD), which prevents PKARIα interaction with AKAPs, with 50-fold selectivity over PKARII.^24^ In cells expressing PKARIα(WT), disruption of AKAP binding by RIAD led to a reduction in the immobile fraction of PKARIα to levels comparable with PKARIα(C17S)-expressing cells (Figure 3D and 3E). The effect of RIAD was present only in PKARIα(WT)-expressing cells, with no significant effect of RIAD on cells expressing the “redox dead” RIα (C17S) mutant (Figure 3E). As before, we saw no effect of the C17S mutant or RIAD on the diffusion rates of the mobile fraction of RIα (Figure IIIC in the Data Supplement). Thus, the disulfide state of RIα appears to influence the extent to which PKA is anchored within the cell, through AKAP binding, without affecting the diffusion rate of PKARIα’s cytosolic fraction or its catalytic activity.

### PKARIα Disulfide Formation Localizes the Holoenzyme to Lysosomal Microdomains in Cardiomyocytes

**Figure 4.**
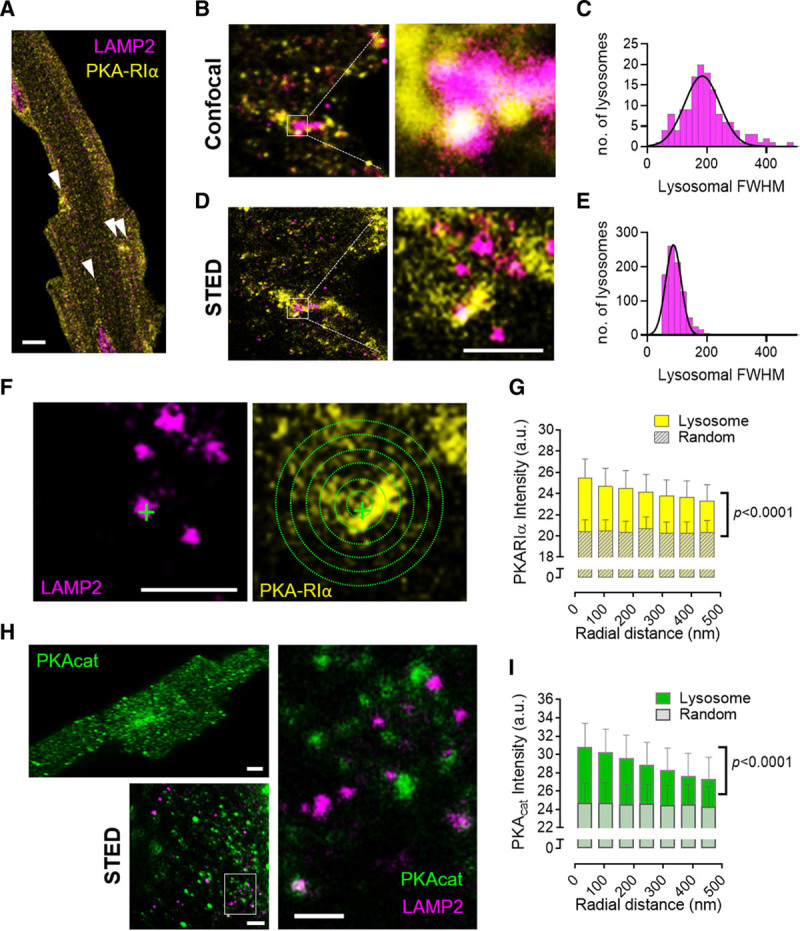
**Both the RIα and PKA_cat_ subunits are found clustered to the lysosome.** **A**, Representative confocal image (scale bar=5 µm) of a cultured WT mouse left ventricular cardiomyocyte immunostained for PKARIα (yellow) and LAMP2 (magenta), showing diffuse staining of endogenous PKARIα as well as punctate regions of staining (arrows). **B**, Magnified confocal images (scale bar=500 nm) indicate that PKARIα clustering occurs near LAMP2-positive lysosomes; however, (**C**) few lysosomes are identified, and their size estimates (full-width half maximum [FWHM]) are overestimated by the resolution of standard confocal microscopy. **D**, Imaging with stimulation emission depletion microscopy (STED) dramatically improves the resolution of LAMP2-positive lysosomes, with (**E**) increased identification and more reliable size estimates (FWHM). **F**, To quantify the degree of PKARIα clustering near lysosomes, as captured in STED mode, LAMP2-positive lysosomes were identified using a custom-build macro in ImageJ (left) and the PKARIα intensity quantified at increasing radial distances (green circles) from each lysosome (right). For comparisons against cytosolically diffuse PKARIα, the same analysis was repeated on each image using random, computer-generated coordinates. Scale bar=500 nm. **G**, Quantification of PKARIα fluorescence intensity in WT adult mouse cardiomyocytes as a function of distance from the lysosome. Fluorescence intensity expressed in arbitrary units (a.u.). Data are mean±SEM; repeated measures 2-way ANOVA with Bonferroni correction, *P*<0.01 for significant interaction between PKARIα fluorescence intensity and distance from the lysosome; n=38 cardiomyocytes, each from 3 mice. **H**, The degree of clustering between PKA_cat_ (green) and LAMP2-positive lysosomes (magenta) was also assessed by STED imaging in WT cardiomyocytes using the same method as in **F**. Scale bars=5 µm for whole cell confocal and 1 µm for magnified STED images. **I**, Quantification of PKA_cat_ fluorescence intensity as a function of distance from the lysosome. Fluorescence intensity expressed in arbitrary units (a.u.). Data are mean±SEM; repeated measures 2-way ANOVA with Bonferroni correction, *P*<0.01 for significant interaction between PKA_cat_ fluorescence intensity and distance from the lysosome; n=35 cardiomyocytes, each from 3 mice. PKA indicates protein kinase A; PKA_cat_, protein kinase A catalytic subunit; PKARIα, regulatory subunit Iα-containing protein kinase A; and WT, wild-type.

If PKARIα disulfide formation affects AKAP-mediated intracellular anchoring, then the disulfide state would also be expected to influence PKARIα subcellular compartmentation; however, to date, the identity of these compartments has remained elusive. Taking advantage of the oxidizing conditions of cell culture, which was shown to induce near-complete PKARIα disulfide formation, we determined whether the disulfide state influenced PKARIα subcellular compartmentation in cardiomyocytes using immunofluorescence imaging in cultured WT and KI LV cardiomyocytes. In WT cardiomyocytes, PKARIα was found to colocalize with mitochondria, the nucleus (Figure IVA in the Data Supplement), and LAMP2-positive lysosomes (Figure 4A and 4B). Whereas confocal microscopy was sufficient to demonstrate that localization to the mitochondria and nucleus was unaffected by the loss of PKARIα disulfide formation in KI cardiomyocytes (Figure IVB in the Data Supplement), superresolution stimulation emission depletion microscopy was required to quantify the extent of PKARIα association with the lysosome and assess the redox dependence of this interaction.

**Figure 5.**
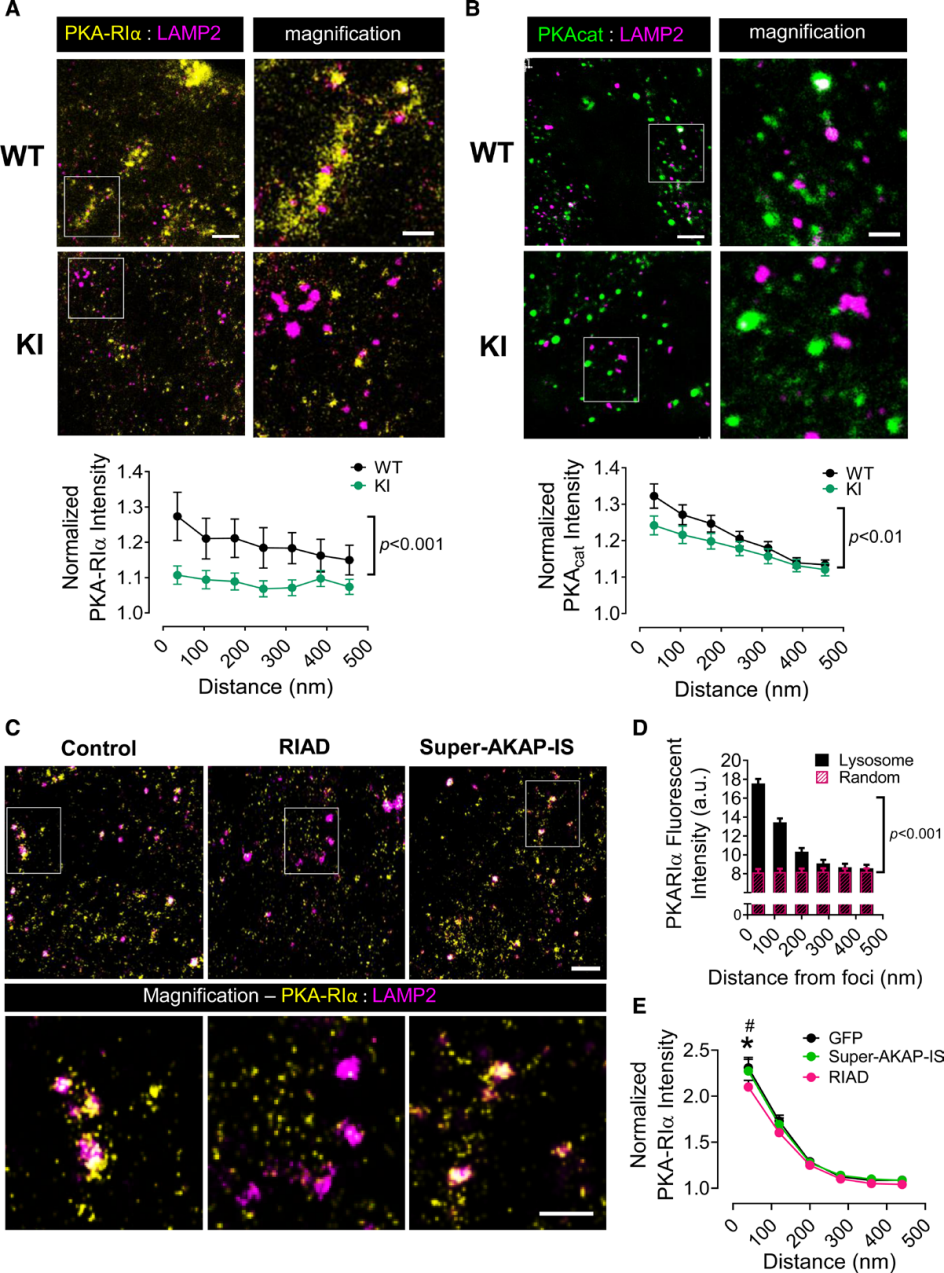
**PKARIα shows AKAP-mediated lysosomal clustering when in the disulfide state.** **A**, **Top**, Stimulation emission depletion microscopy (STED) imaging of PKARIα (yellow) and LAMP2 (magenta) immunostaining in cardiomyocytes isolated from WT or KI mice. **Bottom**, Quantitative comparison of PKARIα intensity in WT and KI cardiomyocytes, normalized for each cell to the cytosolically diffuse intensity, plotted as a function of the radial distance from the lysosome. Normalized data for WT cells were calculated from those included in Figure 4G. Data shown as mean±SEM; repeated measures 2-way ANOVA with Bonferroni correction, *P*<0.001 for significant interaction between genotypes and distance; n=38 to 41 cardiomyocytes, each from 3 mice/genotype. **B**, **Top**, STED imaging of PKA_cat_ (green) and LAMP2 (magenta) immunostaining in cardiomyocytes isolated from WT or KI mice. **Bottom**, Quantitative comparison of normalized PKA_cat_ intensity in WT and KI cardiomyocytes as a function of the radial distance from the lysosome. Normalized data for WT cells were calculated from those included in Figure 4I. Data shown as mean±SEM; repeated measures 2-way ANOVA with Bonferroni correction, *P*=0.005 for significant interaction between genotypes and distance; n=35 to 37 cardiomyocytes, each from 3 mice/genotype. **C**, Neonatal rat ventricular myocytes were transfected for 24 hours with GFP only (transfection control), GFP + the RI α anchoring disruptor, (RIAD) or GFP + the RII anchoring disruptor, super-AKAP-IS, and then fixed and coimmunostained for PKARIα (Atto-647, yellow) and LAMP2 (AF-594, magenta). STED images were acquired only in GFP-positive cells. **D**, PKARIα fluorescent intensity (arbitrary units [a.u.]) was quantified within a given distance from each lysosome as in Figure 4F (binned by Atto-647 resolution; 80 nm). *P*<0.0001 for significant interaction, *P*<0.001 for difference between foci at 80, 160, and 240 nm distance determined by post-hoc testing. **E**, Comparison of normalized intensities within 80 nm of LAMP2-positive vesicles was made between cells transfected with GFP only and those transfected with GFP + RIAD or GFP + super-AKAP-IS. Normalized data for the GFP group were calculated from cells used in Figure 5D. Data shown as mean±SEM; repeated measures 2-way ANOVA with Bonferroni correction, *P<0.001 for GFP versus GFP + RIAD and #*P*<0.01 for GFP + RIAD versus GFP + super-AKAP-IS; n=28 to 38 cardiomyocytes, each from 3 independent isolations/conditions. For **A** through **C**, scale bar=1 μm and 500 nm for all magnified images. AKAP indicates A-kinase anchoring protein; GFP, green fluorescent protein; KI, knock-in; PKA, protein kinase A; PKA_cat_, protein kinase A catalytic subunit; PKARIα, regulatory subunit Iα-containing protein kinase A; and WT, wild-type.

Stimulation emission depletion imaging allowed accurate identification of lysosomes (Figure 4C through 4E)—whose average diameter is less than the 200-nm resolution of standard confocal imaging—and significantly improved quantification of PKARIα fluorescence intensity at nanometer distances.^25^ By quantifying PKARIα fluorescence intensity at increasing radial distances from lysosomal foci (Figure 4F; radial increments=70 nm as determined in Figure VA in the Data Supplement) and comparing that with the PKARIα fluorescence intensity measured at randomly generated coordinates, we were able to demonstrate significant clustering of PKARIα to within 70 nm of lysosomes in WT cardiomyocytes (Figure 4G, Figure VB in the Data Supplement). Using the same approach, we found that PKA_cat_ also clustered near lysosomes (Figure 4H and 4I), indicating that the entire holoenzyme complex was present in the lysosomal microdomain when PKA was highly oxidized. By contrast, in the absence of disulfide formation (ie, KI cardiomyocytes), PKARIα no longer clustered near lysosomes (Figure 5A). Clustering of PKA_cat_ was also found to be reduced in KI cardiomyocytes, albeit to a lesser extent (Figure 5B; *P* value for significant interaction=0.009). The loss of PKA clustering to the lysosome, however, did not appear to affect gross lysosomal distribution (Figure VC in the Data Supplement).

We next assessed whether clustering of PKARIα to the lysosomal microdomain was mediated by AKAP binding. For this, stimulation emission depletion imaging experiments were repeated using the RIAD disruptor peptide in neonatal rat ventricular myocytes, which are more easily cultured and transfected than adult mouse cardiomyocytes. As with adult LV cardiomyocytes, control-transfected neonatal rat ventricular myocytes showed a high degree of PKARIα clustering to LAMP2-positive lysosomes (Figure 5C and 5D). RIAD transfection significantly reduced this colocalization—particularly at the nearest measurable distance—whereas transfection of cells with SuperAKAP-IS, a potent and specific disruptor of PKA-RII:AKAP interactions, did not (Figure 5C and 5E).

Collectively, these data provide strong evidence that induction of PKARIα disulfide formation facilitates localization of the holoenzyme complex to the lysosome of cardiomyocytes in a manner that is AKAP-dependent.

### Intracellular Ca^2+^ Release Is Regulated by PKARIα Through Its Interaction With the Lysosomal TPCs

Lysosomes are known to couple with the mitochondria^26^ and the cardiomyocyte SR,^27^ forming structural microdomains through which lysosomal Ca^2+^ release may affect Ca^2+^ handling by these organelles.^26,28^ We initially assessed, therefore, whether loss of lysosomal-localized PKARIα in KI cardiomyocytes affected mitochondrial or SR Ca^2+^ handling (eg, release and reuptake) under steady-state conditions. As before, we found that the conditions required to assess intracellular Ca^2+^ handling in WT cardiomyocytes led to near-complete induction of PKARIα disulfide formation (94.5±2.3%; as reported in Table II in the Data Supplement).

Direct measurement of mitochondrial Ca^2+^ handling (at 37°C) in permeabilized cardiomyocytes loaded with Rhod-2 showed equivalent levels of mitochondrial Ca^2+^ loading in WT and KI cells challenged with 100 nmol/L free [Ca^2+^] (Figure VIA and VIB in the Data Supplement). Likewise, similar rates of mitochondrial Ca^2+^ efflux were observed between genotypes (Figure VIC in the Data Supplement), indicating that mitochondrial Ca^2+^ handling was unaffected by PKARIα displacement from the lysosome.

**Figure 6.**
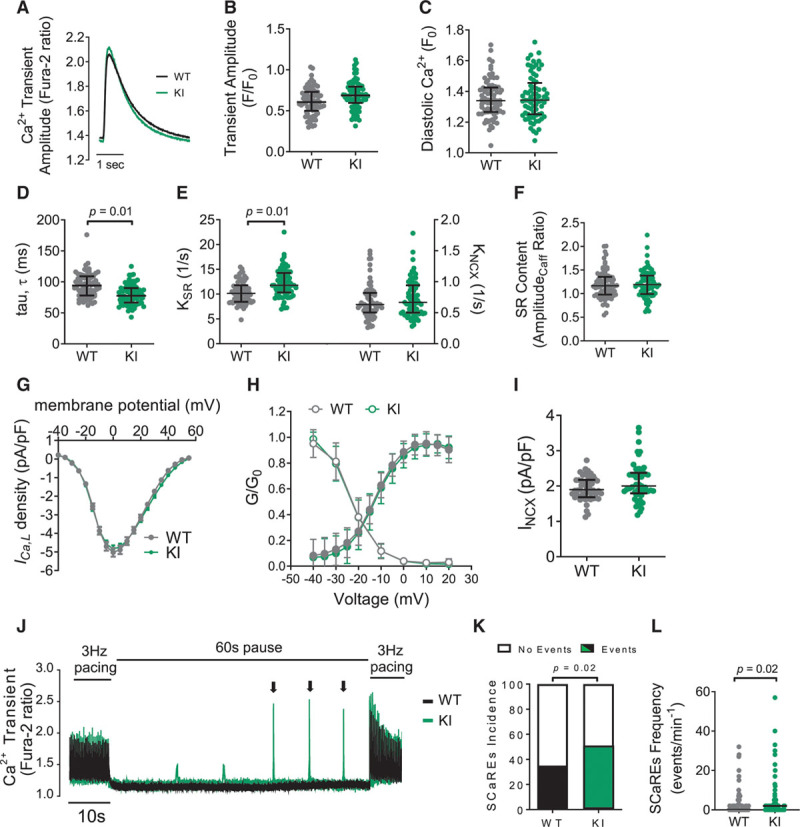
**KI cardiomyocytes display spontaneous Ca^2+^ release in the absence of changes in SR Ca^2+^ load.** **A**, Electrically stimulated intracellular Ca^2+^ ([Ca^2+^]_i_) transients (3 Hz, 35±1°C) showed that, compared with WT, KI cardiomyocytes have similar (**B**) [Ca^2+^]_i_ transient amplitude and (**C**) diastolic Ca^2+^, but have (**D**) a significantly faster [Ca^2+^]_i_ decay rate (tau). **E**, Calculated rate constants for [Ca^2+^]_i_ decline attributed to sarcoplasmic/endoplasmic reticulum Ca^2+^ ATPase (SERCA or K_SR_) (**left**) and sodium/calcium exchanger (NCX or K_NCX_) (**right**) show that the faster rate of [Ca^2+^]_i_ decay in KI cardiomyocytes is driven by faster SERCA-mediated Ca^2+^ uptake, in the absence of measurable difference in total SR Ca^2+^ load (**F**). All data points are shown, with median and interquartile range indicated; statistical analysis was done using a hierarchical model on normally distributed data. Where data were non-normally distributed, logarithmic transformation was applied before statistical testing; n=8 animals per group, 72 cardiomyocytes per group. Patch-clamping of isolated left ventricular cardiomyocytes showed no difference in (**G**) the peak L-type calcium current (*I*_*Ca,L*_), (**H**) *I*_*Ca,L*_ activation (closed circles) or inactivation (open circles) kinetics, or (**I**) the NCX peak current (I_NCX_, taken at –40 mV). n=50 cardiomyocytes, each from 6 mice/genotype. Mann-Whitney test (**I**); repeated measures 2-way ANOVA (**G** and **H**). (**J**) Pace-pause protocol used to assess spontaneous Ca^2+^ release events (SCaREs; indicated by arrows) in WT and KI cardiomyocytes. (**K**) Percentage of cardiomyocytes that developed spontaneous Ca^2+^ release events during a 60 second pause from pacing and (**L**) the frequency of events within the 60 secs. Fisher exact test for panel K and Mann-Whitney test for panel L; n=110 to 112 cardiomyocytes, each from 6 mice/genotype. KI indicates knock-in; SR, sarcoplasmic reticulum; and WT, wild-type.

**Figure 7.**
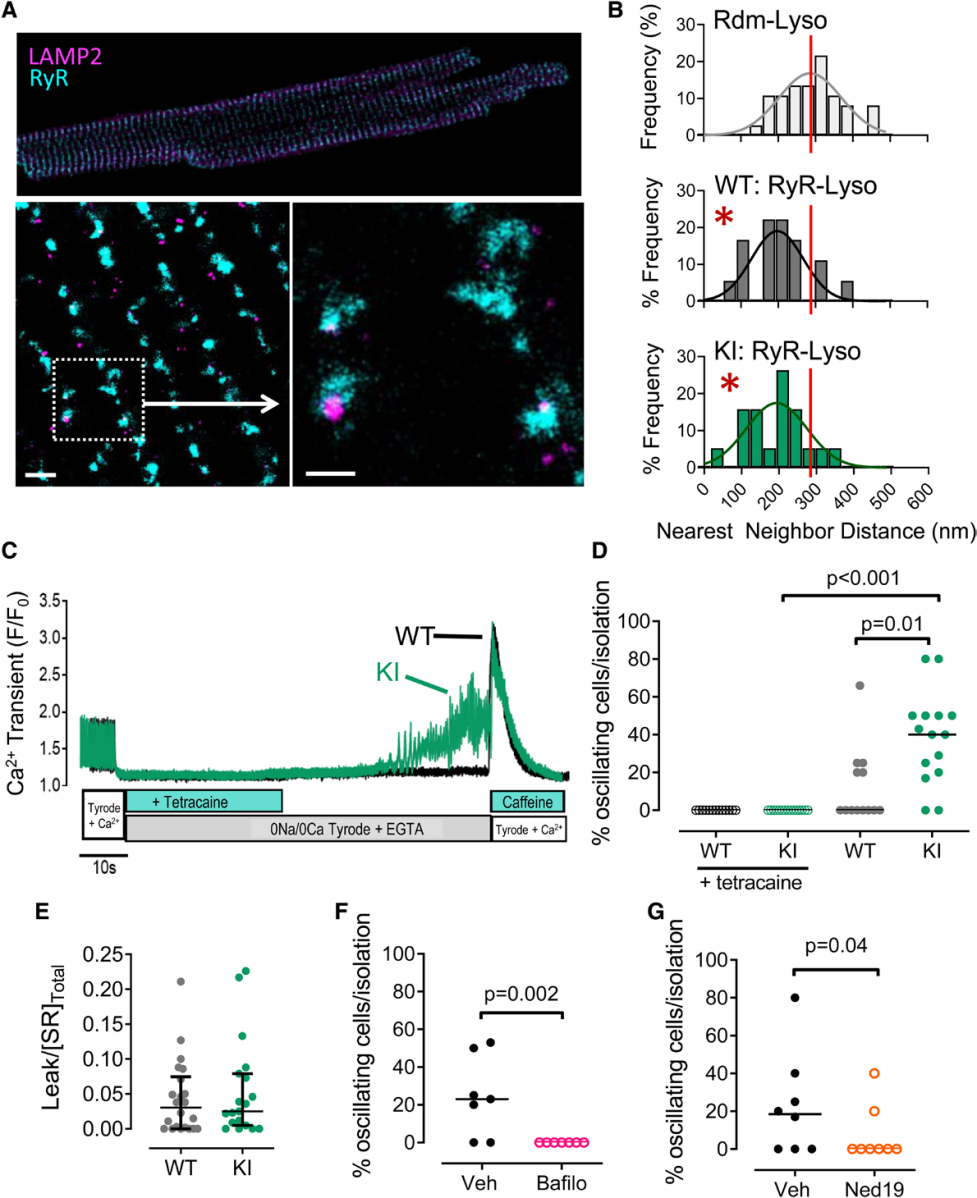
**KI cardiomyocytes display global SR Ca^2+^ oscillations triggered by 2-pore channel–mediated lysosomal Ca^2+^ release.** **A**, Whole cell confocal image of a representative adult mouse left ventricular (LV) cardiomyocyte coimmunostained for RyR2 (cyan) and LAMP2 (magenta). Imaging in high-resolution stimulation emission depletion microscopy mode shows close proximity between LAMP2-positive lysosomes and RyR. Scale bars=1 μm (left zoomed image) and 500 nm (right zoomed image). **B**, RyR-LAMP2 nearest-neighbor distance distributions in WT and KI cardiomyocytes calculated by measuring the distances from the center of the LAMP2-positive lysosome (or a random, computer-generated coordinate, as is the case for the Rdm-Lyso histogram) to the center of the nearest RyR cluster. n=18 to 19 cardiomyocytes, each from 3 mice/genotype. Statistical comparison of experimental (WT or KI) versus Rdm-Lyso distributions using a Mann-Whitney nonparametric test, *P<0.0001. **C**, Protocol used to assess [Ca^2+^]_i_ dynamics and RyR leak simultaneously for **C** through **G**, with trace showing the development of Ca^2+^ oscillations in a KI cardiomyocyte perfused with a modified Tyrode solution containing 0 Na^+^ and 0 Ca^2+^. **D**, The percentage of WT and KI cardiomyocytes per isolation that developed Ca^2+^ oscillations in the presence or absence of tetracaine (10 mmol/L). The Kruskal-Wallis test was used to assess differences between groups; n=13 (WT) and 15 (KI) mice per group. Individual data and their median value are shown. **E**, Oscillations occurred in the absence of differences in RyR leak, quantified by the RyR leak/SR Ca^2+^ load relationship in WT and KI mouse LV cardiomyocytes. All data points shown with median and interquartile range; Mann-Whitney nonparametric test; n=24 cardiomoycytes, each from 6 or 7 mice/genotype. The occurrence rate of Ca^2+^ oscillations was also assessed in cardiomyocytes treated with vehicle (dimethyl sulfoxide) versus (**F**) bafilomycin A1 (Bafilo; 100 nmol/L), or (**G**) Ned-19 (5 μmol/L). Fisher exact test; n=7 mice per group (Bafilo), n=9 mice per group (Ned-19). KI indicates knock-in; Rdm, random foci; RyR, ryanodine receptor; SR, sarcoplasmic reticulum; and WT, wild-type.

Under steady-state pacing (3 Hz; 35±1°C), fura-2–loaded cardiomyocytes showed no significant differences in the intracellular Ca^2+^ transient amplitude or diastolic intracellular Ca^2+^ levels between genotypes, although a mild increase in the rate of intracellular Ca^2+^ decay was observed in KI cardiomyocytes (Figure 6A through 6D). Derivation of the sarcoplasmic/endoplasmic reticulum Ca^2+^ ATPase (SERCA) and Na^+^/Ca^2+^ exchanger–dependent rate constants for free intracellular Ca^2+^ decay indicated a mild enhancement of SERCA-dependent uptake of Ca^2+^ into the SR in KI cardiomyocytes (Figure 6E), independent of phospholamban phosphorylation or an altered phospholamban:SERCA ratio (Figure VIIA through VIID in the Data Supplement). However, measurement of total SR Ca^2+^ content, using rapid caffeine application, showed no genotype-dependent differences (Figure 6F), indicating that the modest increase in SERCA-mediated Ca^2+^ reuptake had no significant effect on SR Ca^2+^ loading under these conditions. In agreement with these findings, echocardiographic parameters of LV function were similar in both genotypes (as reported in Table I in the Data Supplement). Equally, we saw no genotype differences in fractional shortening or the rate of relaxation of isolated cardiomyocytes (Figure VIIE through VIIG in the Data Supplement) in the peak and kinetics of the L-type Ca^2+^ current (Figure 6G and 6H) or in the Na^+^/Ca^2+^ exchanger current (Figure 6I). Nevertheless, KI cardiomyocytes displayed a higher incidence of spontaneous Ca^2+^ release events during a pause from steady-state pacing (Figure 6J through 6L), suggesting that the displacement of PKARIα from the lysosomal microdomain in KI cardiomyocytes may be leading to dysregulated lysosomal Ca^2+^ release sufficient to directly trigger ryanodine receptor (RyR) opening. For this to occur, close physical proximity between the 2 structures would have to take place. Stimulation emission depletion imaging confirmed that LAMP2-positive lysosomes were closely coupled with RyRs (Figure 7A and 7B), with nearest-neighbor distance histograms in WT and KI cardiomyocytes showing the majority of the lysosomes lying in close (ie, <200 nm) proximity to RyRs, with no significant difference between genotypes (Figure 7B).

We therefore assessed the dynamics of intracellular Ca^2+^ release from RyR by perfusing WT or KI cardiomyocytes with a 0Na^+^/0Ca^2+^ extracellular solution (which prevents triggering of RyR opening from extracellular sources) and included the use of the reversible RyR inhibitor, tetracaine, to allow for simultaneous quantification of intrinsic RyR Ca^2+^ leak. Cardiomyocytes showed stable Ca^2+^ transient recordings under tetracaine perfusion, with no spontaneous events occurring in either genotype under these conditions. However, following tetracaine washout, a significantly high proportion of KI cardiomyocytes developed dramatic Ca^2+^ oscillations (Figure 7C and 7D). No differences in the RyR leak/load relationship (an indirect assessment of RyR opening probability^29^; Figure 7E) or PKA-mediated RyR phosphorylation (Figure VIIH in the Data Supplement) were found between genotypes, indicating that the Ca^2+^ oscillations were unlikely to be driven by inherent changes in RyR opening. By contrast, depletion of lysosomal Ca^2+^ stores using acute bafilomycin A1 treatment completely abolished Ca^2+^ oscillations under 0Na^+^/0Ca^2+^ conditions (Figure 7F), whereas competitive inhibition of Ca^2+^-permeable lysosomal TPCs using Ned-19 significantly attenuated the incidence of Ca^2+^ oscillations (Figure 7G). Measurement of SR Ca^2+^ load (in nonoscillating cells) indicated that the ability of either drug to prevent global SR Ca^2+^ oscillations was not a consequence of reduced SR Ca^2+^ content (Figure VII I and VIIJ in the Data Supplement), supporting the conclusion that these events were a direct result of spontaneous lysosomal Ca^2+^ release from TPCs, occurring when PKARIα was no longer localized to the lysosome.

Despite the presence of spontaneous SR Ca^2+^ release in KI cardiomyocytes, we did not observe an increase in pacing-induced ventricular arrhythmias in these mice (Figure VIIIA in the Data Supplement). Likewise, there was no evidence for induction of Ca^2+^-activated stress responses in KI hearts. Specifically, transcript levels for multiple markers of the unfolded protein response—a conserved system of endoplasmic reticulum stress signaling cascades activated in response to protein misfolding or altered SR/endoplasmic reticulum Ca^2+^ content—showed no evidence of increased transcriptional activation in KI LVs (Figure VIIIB in the Data Supplement). KI LVs also showed no marked difference in the conversion of LC3-I to LC3-II or degradation of p62 (Figure VIIIC in the Data Supplement), which, together, indicated that activation of the autophagosome-lysosome pathway was not altered in these mice.

### Redox-Dependent Regulation of Lysosomal Ca^2+^ Release by PKARIα Is Cardioprotective Against I/R Injury

**Figure 8.**
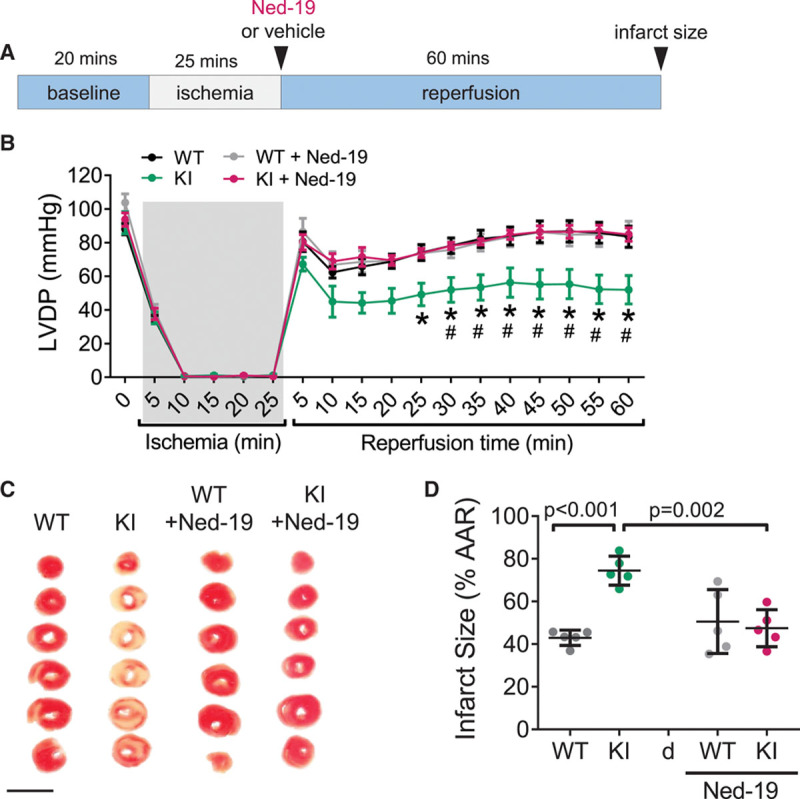
**Disulfide-modified PKARIα protects the myocardium from I/R injury through its regulation of lysosomal Ca^2+^ release.** **A**, After baseline stabilization, WT and KI hearts were subjected to 25 minutes of global ischemia and 60 minutes of reperfusion. Either 10 µmol/L Ned-19 or an equivalent volume of dimethyl sulfoxide (vehicle) was included in the reperfusion solution. **B**, Compared with WT hearts, KI hearts showed worse left ventricular developed pressure (LVDP) recovery following I/R, which was prevented by Ned-19 treatment. Data are mean±SEM with statistical comparisons made using 2-way ANOVA with Bonferroni correction for pairwise comparison, *P<0.01 for KI versus WT, #*P*<0.01 for KI versus KI + Ned-19; n=7 to 9 hearts per group. **C** and **D**, Post-I/R infarct size, assessed by TTC staining and quantified as the percentage of area at risk (% AAR), was larger in KI hearts compared with WT; this difference was abolished by adding Ned-19 in the reperfusion solution. For **C**, scale bar=1 cm. All data points are shown with mean±SD indicated; 1-way ANOVA with Bonferroni correction; n=5 hearts per group. I/R indicates ischemia/reperfusion; KI, knock-in; PKARIα, regulatory subunit Iα-containing protein kinase A; TTC, Triphenyltetrazolium chloride; and WT, wild-type.

SR Ca^2+^ oscillations are known to occur in the initial period of myocardial reperfusion, leading to cell death and LV dysfunction.^30^ Given our observation that PKARIα disulfide formation is induced shortly after myocardial reperfusion in humans and mice, we posited that inhibition of global Ca^2+^ release by oxidized, lysosomally targeted PKARIα may confer cardioprotection in the postischemic heart. To test this hypothesis, hearts from WT and KI mice were subjected to ex vivo I/R, with LV function measured throughout and infarct size assessed following the 60 minutes reperfusion (Figure 8A). Furthermore, to determine the contribution of TPC-dependent lysosomal Ca^2+^ release, hearts of either genotype were administered Ned-19 (or dimethyl sulfoxide vehicle) at the time of reperfusion.

Although no difference in LV hemodynamic measurements were seen during the baseline stabilization period (Table III in the Data Supplement), KI hearts administered vehicle at reperfusion showed significantly lower LV developed pressures throughout the reperfusion period (Figure 8B) and displayed 2-fold larger infarcts compared with WTs (Figure 8C and 8D). Absence of differences in PKA-dependent RyR phosphorylation during I/R (Figure IX in the Data Supplement) ruled out the possibility that direct alterations in RyR accounted for the poorer outcome in vehicle-treated KI mice. Instead, inhibition of lysosomal Ca^2+^ release, by addition of Ned-19 at the time of reperfusion, was sufficient to restore both contractile function and infarct size in KI hearts to levels comparable with WT, with no further protective effects observed in WT hearts. These findings are consistent, therefore, with a model in which disulfide-modified PKARIα limits I/R-induced Ca^2+^ overload by decreasing lysosomal triggering of global SR Ca^2+^ release.

## Discussion

Our findings led us to 3 major conclusions: (1) PKARIα disulfide formation is a consistent and conserved response to myocardial I/R injury in vivo, occurring both in humans and mice; (2) oxidation of PKARIα serves as a means to compartmentalize PKARIα within the lysosomal microdomain, where it acts as an inhibitor of TPC-dependent Ca^2+^ release; and (3) this regulatory mechanism is an adaptive response to I/R, which allows the heart to limit the extent of injury and aid functional recovery.

### Functional Impact of PKARIα Disulfide Formation on Kinase Function and Localization

Although redox modification of several kinases has been shown to promote catalytic activation,^21^ our data rule out the possibility that PKARIα disulfide formation has the same effect. Instead, we provide strong evidence that the principal regulatory function of PKARIα disulfide formation is to promote localization of the holoenzyme complex to distinct subcellular compartments through enhanced AKAP binding. This conclusion is at odds with some previous studies, which report increased PKA activity in response to elevations in ROS^31,32^ and reactive nitrogen species.^33^ However, the observed changes in PKA catalytic activity were inferred from downstream functional readouts—some of which have important limitations^34^—or increased substrate phosphorylation, which make it difficult to distinguish between genuine increases in PKA catalytic activity versus focused subcellular targeting of the enzyme. The use of a genetically encoded FRET biosensor here, in conjunction with PKARIα KI cardiomyocytes, provided a robust means to directly assess changes in intrinsic catalytic activity with varying degrees of PKARIα disulfide formation, and showed no correlation between the 2, suggesting that previous findings may instead be a consequence of substrate-induced activation within specific microdomains,^35^ a characteristic unique to RIα-containing PKA.

The observation that physically restricted pools of PKARIα are lost when disulfide formation is prevented (C17S mutation) argues strongly for a concomitant loss of AKAP-mediated anchoring of PKARIα that is dependent, at least in part, on the structural stability afforded by the disulfide bond.^22^ In support of this hypothesis, disruption of RIα-AKAP interaction in fluorescence recovery after photobleaching experiments caused significant liberation of PKARIα from the immobile pool, an effect that was not observed in C17S-expressing cells. Likewise, in cardiomyocytes both the C17S “redox dead” mutation and disruption of RIα-AKAP binding using RIAD resulted in displacement of the PKARIα holoenzyme from lysosomes. For the latter, lysosomal localization of PKARIα was shown to be diminished, but not abolished, by RIAD disruption, a finding that may reflect suboptimal concentrations of transfected RIAD in the cells because of some degree of peptide degradation, as reported previously.^24^

### Regulation of Lysosomal Ca^2+^ Release by PKARIα

Oxidation-dependent localization of PKARIα to the lysosome represents a significant, and entirely novel, mechanism through which PKA regulates Ca^2+^ release in the heart. Our classic understanding of Ca^2+^ regulation by PKA involves the rapid phosphorylation of key Ca^2+^ handling proteins, including RyR, phospholamban, and the L-type Ca^2+^ channel, which act concordantly to enhance contraction and relaxation. However, several studies suggest that these substrates are uniquely targeted by RII-containing pools of PKA, whereas activation of PKARIα has little effect on excitation-contraction coupling.^36–38^ Consistent with this, we find no evidence for differential phosphorylation of “classic” PKA targets (eg, in RyR or phospholamban), nor do we see differences in cardiomyocyte contractility or LV systolic function in vivo or in isolated hearts between WT and KI mice. Instead, we find that the striking Ca^2+^ oscillations and spontaneous Ca^2+^ release events observed in KI cardiomyocytes are a result of dysregulated Ca^2+^ release from lysosomal TPCs when PKARIα is absent from this microdomain.

Lysosomal Ca^2+^ efflux can promote SR Ca^2+^ release either by triggering RyRs Ca^2+^ release directly^39^ or by enhancing SR Ca^2+^ loading.^28^ We observed minimal enhancement of SERCA-mediated Ca^2+^ uptake in KI cardiomyocytes, with no obvious difference in SR Ca^2+^ load or RyR leak compared with WT. Instead, we found spontaneous triggering of SR Ca^2+^ release (in the absence of sarcolemmal Na^+^/Ca^2+^ flux), which could be prevented by inhibiting RyR opening or lysosomal Ca^2+^ release through TPC channels. These findings, and the fact that we found lysosomes lying in close physical proximity to the RyR, indicate that PKARIα directly modulates the crosstalk between lysosomal TPCs and RyRs. Of note, the prevention of SR Ca^2+^ release by these drugs was not driven by a reduction in SR Ca^2+^ load. In fact, in KI cardiomyocytes, Ned-19 significantly increased load. Because this would be expected to promote Ca^2+^ oscillations by increasing RyR opening probability, it is not likely that the increased load had a direct effect on the inhibitory effects of Ned-19.

### PKARIα Disulfide Formation as an Adaptive Response to I/R

Although early bursts of ROS are thought to be the primary mediators of reperfusion-induced injury,^1,2^ evidence indicates that some degree of ROS are needed at the time of reperfusion to protect the heart.^4,5^ In particular, I/R-induced elevations in NADPH oxidase (NOX)-derived ROS activate redox signaling pathways that promote cell survival.^3,40,41^ In this regard, compartmentation of oxidant sources and their downstream targets is suggested to be a discriminating factor between adaptive versus maladaptive signaling.^3^ Consistent with this, we observed that disulfide-dependent compartmentation of PKARIα was a necessary event to confer cardioprotection. When this response was lost in KI mice, cardiomyocytes exhibited dysregulated lysosomal Ca^2+^ release, which ultimately led to exacerbated I/R injury.

The fact that pharmacological inhibition of TPCs at the time of reperfusion reduced infarct size and improved functional recovery in KI hearts highlights a causal role for lysosomal Ca^2+^ release in mediating PKARIα’s adaptive response to I/R. Inhibition of TPCs, either pharmacologically or by genetic knockdown, has been shown to protect the heart against I/R injury, both in vitro and in vivo.^42^ It is interesting that pharmacological inhibition of TPC conferred cardioprotection in WT mice only when a more potent derivative of Ned-19, called Ned-K, was used.^42^ This may explain why, in keeping with the same report,^42^ we did not see an added benefit in WT hearts perfused with Ned-19.

Although our observations provide strong evidence for a cardioprotective role of oxidized PKARIα independent of increases in cAMP, it is conceivable that further activation of RIα-containing pools of PKA by cAMP may provide additional protection from I/R injury. Glucagon-like peptide-1 and prostaglandin E1, which are currently being tested in clinical trials for their use in treating myocardial infarction^43,44^ and reperfusion injury,^45,46^ have both been found to promote selective activation of RI- but not RII-containing PKA in cardiomyocytes.^36,38^ Preclinical studies have already shown that the beneficial effects of these hormones rely on PKA,^47,48^ although the downstream mechanisms have yet to be fully elucidated. Our findings indicate that inhibition of lysosomal Ca^2+^ release by PKARIα may contribute to the cardioprotective effects of these hormones and further suggest that targeted enhancement of lysosomal PKARIα, or inhibition of TPCs, offers a novel adaptive signaling pathway to exploit for the prevention of I/R injury.

### Potential Limitations

Although we showed that disulfide formation is important for restricting PKARIα to lysosomal regions, it is not clear from our data whether regulation of lysosomal function occurs through a direct interaction between PKARIα and TPCs or through more distal PKA-dependent signaling events in this microdomain. In vitro PKA can directly phosphorylate TPCs and alter channel opening^49^; whether this occurs in vivo and is influenced by PKARIα disulfide formation remains to be explored. Similarly, we cannot exclude that differences in nicotinic acid adenine dinucleotide phosphate (the ligand for TPC channels) between genotypes may have contributed to our results. However, this seems unlikely, as altered nicotinic acid adenine dinucleotide phosphate levels have been shown primarily to increase SR Ca^2+^ loading and Ca^2+^ transient dynamics in cardiomyocytes,^50^ neither of which was found to be materially different between KI and WT mice. It should also be noted that the contribution of enhanced SR Ca^2+^ oscillations to the exacerbated I/R injury seen in KI mice was inferred from studies in isolated cardiomyocytes, as opposed to a direct assessment during I/R. Nevertheless, our data in KI mice showing that Ned-19 prevents SR Ca^2+^ oscillations in cardiomyocytes and limits myocardial I/R injury strongly support a link between them.

## Conclusions

Our work identifies, for the first time, oxidation-dependent compartmentation of the PKARIα holoenzyme to the lysosomal microdomain, where it acts as a potent inhibitor of intracellular Ca^2+^ release. In the setting of I/R, where PKARIα disulfide formation is induced, this regulatory mechanism is critical for limiting infarct size and offers a novel target for the design of cardioprotective therapeutics.

## Acknowledgments

The authors thank Pablo Hernandez-Varas and Christoffer Langerholm (Wolfson Imaging Center, University of Oxford) for expertise in stimulation emission depletion microscopy; Ricardo Carnicer, Craig Lygate, and Debra McAndrews (University of Oxford) for the provision of left ventricular tissue from mice undergoing in vivo ischemia and reperfusion or sham surgery; and S. Nashitha Kabir (University of Birmingham) for technical assistance.

## Sources of Funding

This work was supported by the British Heart Foundation (CH/12/3/29609 to B.C. and J.N.S.; RG/11/15/29375 to B.C. and R.J.; RG/16/12/32451 to B.C. and B.V.; FS/17/17/32438 to P.G.; RG/17/6/32944 to M.Z. and S.M.; PG/15/5/31110 to M.Z.; RG/15/9/31534 to P.S.; RG/12/5/29576 to K.M.C. and S.C.; PG/17/44/33064 to P.E.; Accelerator Award AA/18/2/34218 to L.F.); the National Institute for Health Research Oxford Biomedical Research Center (to B.C.); the Medical Research Council (MR/P023150/1 to P.E.; MR/S005382/1, MRC/BBSRC/EPSRC, and MR/K01577X/1 to D.W.); the Garfield-Weston Foundation (MPS/IVIMS-11/12–4032 to B.C. and N.R.); and the Wellcome Trust (0998981Z/12/Z to O.L.).

## Disclosures

None.

## Supplemental Materials

Data Supplement Methods

Data Supplement Tables I–III

Data Supplement Figures I–IX

References 51–56

## Supplementary Material


