# Supplementary material for: Oxidation of Protein Kinase A Regulatory Subunit PKARIα Protects Against Myocardial Ischemia-Reperfusion Injury by Inhibiting Lysosomal-Triggered Calcium Release
Source: Circulation. 2020 Nov 13;143(5):449–65. doi: 10.1161/CIRCULATIONAHA.120.046761 (PMC7846288; doi:10.1161/CIRCULATIONAHA.120.046761)
Supplement: Supplementary file 1 [file cir-143-449-s001.pdf]

SUPPLEMENTAL MATERIAL

Oxidation of Protein Kinase A Regulatory Subunit PKARIα protects against myocardial ischemia-reperfusion injury by inhibiting lysosomal-triggered calcium release

Jillian N. Simon, Ph.D.[1,#], Besarte Vrellaku M.Sc.[1], Stefania Monterisi, Ph.D.[2], Sandy M. Chu, Ph.D.[1], Nadiia Rawlings, Ph.D.[1], Oliver Lomas, D.Phil, M.R.C.P.[1], Gerard A. Marchal, M.Sc.[1], Dominic Waithe, Ph.D.[3], Fahima Syeda, Ph.D.[4], Parag R. Gajendragadkar, M.Phil, M.R.C.P.[1], Raja Jayaram, M.D, D.Phil.[1], Rana Sayeed, Ph.D., F.R.C.S.[5], Keith M. Channon, M.D., F.R.C.P.[1], Larissa Fabritz, M.D.[4,6], Pawel Swietach, D.Phil [2], Manuela Zaccolo, M.D., Ph.D.[2], Philip Eaton, Ph.D.[7], Barbara Casadei M.D, D.Phil., F.R.C.P [1]

[1]Division of Cardiovascular Medicine, Radcliffe Department of Medicine, University of Oxford, Oxford, UK.

[2]Department of Physiology, Anatomy and Genetics, University of Oxford, Oxford, UK.

[3]Wolfson Imaging Centre, Weatherall Institute of Molecular Medicine, University of Oxford, Oxford, UK.

[4]Institute of Cardiovascular Sciences, University of Birmingham, Birmingham, UK.

[5]Cardiothoracic Surgery, Oxford Heart Centre, Oxford University Hospitals NHS Foundation Trust, Oxford, UK.,

[6]Department of Cardiology, University Hospitals Birmingham, Birmingham, UK.

[7]William Harvey Research Institute, Barts and The London School of Medicine & Dentistry, Queen Mary University of London, Charterhouse Square, London, UK.

[#]Correspondence:
Jillian N. Simon (jillian.simon@cardiov.ox.ac.uk)
Division of Cardiovascular Medicine, Radcliffe Department of Medicine
University of Oxford
John Radcliffe Hospital
L6, West Wing
Oxford, UK OX3 9DU

Supplemental (Expanded) Methods:

Human atrial myocardium biopsies. In patients undergoing cardiac surgery with cardiopulmonary bypass, a sample of the right atrial appendage was taken at two time-points: 1) before the insertion of the venous cannula in the right atrium and commencement of cardioplegia, and 2) minutes after venous decannulation and cardiac reperfusion. The study was approved by the local Research Ethics Committee (REC reference: 07/Q1607/38) and the institutional review committee and all patients gave written, informed consent.

Animals. "Redox dead" PKARIα knock-in (KI) mice (C57BL/6 background), in which the nucleotides encoding for cysteine at position 17 were mutated to nucleotides encoding for serine (Cys17Ser), were generated as previously described [19]. Only male mice were used for assessment of infarct size. For all other studies, KI mice (12–18 weeks old) of both sexes were compared with their wildtype (WT) littermates. All experiments involving animals were carried out in accordance with the UK Home Office Guidance on the Operation of Animals (Scientific Procedures) Act, 1986 and the guidelines approved by the local Ethics Committee at the University of Oxford.

In vivo model of ischemia-reperfusion (I/R). Ligation of the left anterior descending (LAD) coronary artery was performed as previously described[51], with reperfusion of the coronary artery 45 mins after ischemia (confirmed by direct visualization of blood returning to the infarcted myocardium). Following 30 mins reperfusion, hearts were excised, the remote vs ischemic regions separated and the two regions snap frozen in liquid nitrogen. For sham operations, mice were anesthetized and the chest opened for an equivalent time, but without the surgical intervention.

Immunoblotting. Immunoblotting to assess the extent of PKARIα disulfide formation was performed under non-reducing conditions. Equal amounts of protein (20 μg) was electrophoresed in 12% Criterion TGX gels (BioRad), transferred to 0.2 µm nitrocellulose membrane, and probed overnight at 4°C with a Regulatory Subunit Type-1 (RI)-specific PKA antibody (BD Transduction). All other immunoblotting was performed in the presence of reducing agents, using the abovementioned conditions.

Echocardiography. Mice were anaesthetised using an initial 5% isoflurane/oxygen mix followed by maintenance using a 2% isoflurane/oxygen mixture and placed on a heated table with integrated ECG monitoring capabilities. An appropriate depth of anaesthesia was maintained and echocardiography performed using a VisualSonics Vevo 2100 system and an 18-38 MHz MS400 transducer probe (FUJIFILM VisualSonics Inc, Toronto). Left ventricular (LV) mass and systolic function was assessed at near-physiological heart rates (~450bpm). All measurements were taken using M-mode echocardiography in a short axis view at the level of the papillary muscles.

cAMP-affinity capture. Left ventricular (LV) tissue homogenates (600 µg in 500 µL lysis buffer) were combined with 50 µL agarose beads coated with Sp-2-AEA-cAMPS-Agarose (BioLog) and incubated for 2h at 4°C with gentle rotation. Beads were then gently pelleted and washed 5 times with ice-cold phosphate buffered saline and then capture proteins were eluted by boiling samples for 10 mins at 96°C in 2x SDS-loading

buffer. For controls, LV homogenates were pre-incubated for 30 mins with either 60 µmol/L cAMP or cGMP prior to affinity capture.

Quantitative real-time polymerase chain reaction analysis. Total transcript levels were measured, using technical duplicates, in WT and KI LV homogenates using quantitative reverse-transcriptase PCR (qRT-PCR). TaqMan Gene Expression Assays for calcineurin/NFAT or unfolded protein response (UPR) target genes (Applied Biosystems; assay IDs: mouse Atf4 Mm00515325_g1; mouse Atf 6 Mm01295319_m1; mouse CHOP Mm01135937_g1; mouse Xbp1 Mm00457357_m1; mouse Bip Mm00517691_m1; mouse Nppa Mm01255748_g1; mouse Nppb Mm01255770_g1; mouse Actn1 Mm01304396_m1; mouse Actn2 Mm00473657_m1; mouse Myh7 Mm00600555_m1; mouse Rcan1.4 Mm00627762_m1; mouse PPIA Mm02342430_g1) were used to quantify expression levels of each transcript.

Adult mouse LV cardiomyocyte isolation. Adult mouse LV cardiomyocytes were isolated from either PKARIα KI or WT littermates, aged 12-18 weeks, using a standard enzymatic dispersion technique as previously described[52].

***Neonatal rat cardiomyocyte (NRVM) isolation*.** Primary NRVMs were obtained from 1 to 2 day old Sprague-Dawley rats. Cells were isolated using enzymatic digestion and a 'pre-plating' step was implemented to remove fibroblasts from the myocute-enriched supernatant. Cells were cultured for 24 hours in M1 medium (DMEM High Glucose, MEM199, Horse serum, New born Calf serum, Glutamine, Pen/Strep) and then switched to a serum-free medium containing DMEM High Glucose, MEM199, Insulin-Transferring-Selenium-X Supplement, Glutamine, Pen/Strep. NRVMs were transfected with Transfectin Lipid Reagent (BioRAD), following the supplier's instructions.

Adenoviral infection. Adenoviral infection of genetically encoded fluorophores was carried out as described previously described[38]. A replication-deficient adenoviral pDUAL vector sub-cloned with the AKAR3ev was generated and given as a gift by Dr K Lefkimmiatis (Oxford, UK). Amplification and purification of the viral construct was provided by Vector Biolabs (Malvern, PA, USA) at a titer of 10^{10} particle forming units (PFUs) per ml. After cardiomyocyte isolation and washing, cells were cultured for two hours on laminin-coated glass coverslips in Modified Eagles Medium (MEM) supplemented with 2.5% fetal bovine serum. Once the cells had adhered to the coverslips, the medium was replaced with MEM alone and incubated for three hours with virus at a ratio of 100 viral particles to one cardiomyocyte. After incubation, the cells were washed with MEM three times and supplemented with Cytochalasin D to maintain cardiomyocyte structural integrity in culture.

Fluorescence resonance energy transfer (FRET) imaging. *E*xperiments were performed 24 hours after infection of cardiomyocytes. Cells were maintained at room temperature in Tyrode buffer solution with 1.4mM Ca^{2+} and imaged on an inverted microscope (Nikon Ti-S) using a CFI Plan Fluor (40X N.A. 1.30) oil immersion objective. The microscope was equipped with a coolSNAP HQ monochrome camera system (Photometrics, USA) and a beam-splitter for the recording of dual wavelength emission of YFP and CFP (Dual-view simultaneous-imaging system, DV2 mag biosystem, Photometrics, USA). The FRET filter settings used throughout were: CFP excitation filter ET436/20x, dichroic mirror 455DCLP (Chroma Technology, Germany)

in the microscope filter cube; dichroic mirror 505DCLP, YFP emission filter 545 nm, and CFP emission filter 480 nm (Chroma Technology, Germany) in the beam splitter. Images were acquired using MetaFluor Software, (Cairn, UK) and fluorescence emission intensity on excitation at 430 nm expressed as R/R_0 where R is the intensity at time t and R_0 is the intensity at baseline mean of 120 seconds. Forskolin (25µmol/L), an activator of adenylyl cyclase, and IBMX (3-isobutyl-1-methylxanthine; 100 µmol/L), a specific but non-selective inhibitor of phosphodiesterases that hydrolyse cAMP were used to confirm that the AKAR3ev sensor was functional at the end of each experiment. Cells which did not show a response to Forskolin + IBMX were excluded from the data analysis.

Disruptor peptide constructs. pcDNA3.1 (Invitrogen) plasmids encoding for the PKA type-1 (RIAD[24]: LEQYANQLADQIIKEATE) or type-2 (SuperAKAP-IS[53]: QIEYVAKQIVDYAIHQA) anchoring inhibitors were obtained as previously described[38]. For expression of WT or mutant (C17S or H24A) forms of PKARIα, the pCDNA3-mouse PKA-RIalpha-mEGFP (Addgene #45525) was used, with site-directed mutagenesis performed by PCR to generate the single amino acid substitutions. Prior to amplification, DNA constructs were checked by Sanger nucleotide sequencing (SourceBioscience, UK) to confirm the presence of the appropriate mutation. For expression in cells, plasmid DNA was transfected using TransFectin Lipid Reagent (BioRad) according to the manufacturer's protocol. All experiments were carried out 24-48 h after transfection.

Fluorescence recovery after photobleaching (FRAP) imaging. RIα knock-out mouse embryonic fibroblasts (*prkar1a*$^{-/-}$ MEFs[54]) were used to express plasmids encoding WT or the C17S and H24A mutant PKA RIα-GFP and experiments were performed 24-48 hours after transfection using a Zeiss LSM 700 confocal imaging system at 37°C. Fluorescence in GFP-expressing cells was excited at 488 nm and emission was recorded >520 nm using an x40 objective. After baseline fluorescence was collected in the field of view, a photobleaching protocol was applied to an 18.6 µm circular region-of-interest (ROI) within the cytoplasmic area of a cell. Photobleaching pulses of 488 nm laser were delivered until fluorescence intensity dropped to 50% of the baseline, at which point the time course of fluorescence recovery was monitored as a read-out of diffusivity. To normalize for background changes in fluorescence, unrelated to FRAP, the time course in the bleached cell was normalized to the time course obtained concurrently in a distal, unbleached region within the same cell. The time course of fluorescence recovery was best-fitted (least squares method) to a bi-exponential equation to quantify the time-constants (τ) of the 'slow' and 'fast' diffusing species, and their fractional contribution to total fluorescence signal (C_{slow}, C_{fast}), and a residual term (C_{fixed}) that describes the fractional contribution from immobilised proteins:

$$\frac{F}{F_0} = C_{fixed} + C_{slow} \times \left(1 - exp\left(-\frac{t}{\tau_{slow}}\right)\right) + C_{fast} \times \left(1 - exp\left(-\frac{t}{\tau_{fast}}\right)\right)$$

The fitting was constrained so that the sum of C_{slow}, C_{fast} and C_{fixed} was 1, and τ_{slow} was greater than τ_{fast}.

Confocal and super-resolution imaging. Confocal and Stimulation Emission Depletion (STED) images were acquired with a Leica TCS SP8 3X STED system equipped with lasers for depletion of fluorophores emitting in the blue/green (592 nm), orange (660 nm) and red/far-red (775 nm) ranges. Imaging was done on isolated LV cardiomyocytes following 2-4 h of cell culture on laminin-coated coverslips or in NRVMs 48-72 h after plating. Mitochondria were labelled in live adult mouse cardiomyocytes using 30 min incubation (at 37°C) with Mitotracker-Red CMXRos (100 nmol/L; Life Technologies), after which time cardiomyocytes were fixed for 15 mins with 4% PFA, permeabilized for 4 min with 0.5% Triton-X100 (in PBS) and then blocked and stained overnight with anti-PKA-RI (mouse, 1:300; BD Transduction) diluted in 5% donkey serum. Nuclei were stained in the final wash step using NucBlue (1 drop/mL; Thermo). Anti- LAMP2 (rabbit, 1:100; Thermo), anti-PKAcat (mouse, 1:300; clone B-4 Santa Cruz) and anti- RyR2 (mouse, 1:500; Thermo) primary antibodies were also used for co-staining. AF-488 (Donkey anti-mouse; 1:500), AF-594 (Donkey anti-rabbit; 1:500) or Atto-647 (Donkey anti-mouse; 1:500) secondary antibodies were used, as appropriate. For imaging experiments in NRVM, co-transfection of the RIAD or SuperAKAP-IS disruptor peptide with a GFP plasmid was done 24-48 h prior to fixation, and only GFP-positive cells were selected for imaging. A chromatically optimized oil immersion objective (HC PL APO 100X/1.40 OIL STED WHITE, Leica Microsystems) was used for imaging, and a tunable pulsed (80 MHz) super-continuum whitelight fiber-laser emitting from 470 to 670 nm was used for excitation. Selected excitation wavelengths were 488 nm for AlexaFluor488 (depleted by 592 nm STED laser at 25% laser power), 561 nm for AlexaFluor594 (depleted by 775 nm STED laser at 65% laser power) and 633 nm for Atto-647 (depleted by 775 nm STED laser at 75% laser power). For multi-colour STED imaging a pair of depletion wavelengths (775/592) was applied. Fluorescence signals were passed through a dichroic mirror optimized for each STED laser, including notch filters placed in front sensitive photodetectors (Leica Hybrid Detectors, time gated between 1.4 and 7 ns for AF488 emission capture and between 0.5 and 7 ns for AF594 emission capture), and a 1 Airy unit pinhole. Pixel size was fixed at 20 nm. Dual-color images were acquired sequentially frame by frame at a scan speed of 400 lines per second with 4x line accumulation. Images were blindly analysed in ImageJ using the JACoP plugin[55], for calculation of the Pearsons' correlation coefficient, or a custom-made script to measure PKA intensity relative to the lysosome.

Mitochondrial Ca^{2+} measurement. Mitochondrial Ca^{2+} (Rhod-2AM) was measured at 37°C in isolated cardiomyocytes following permeabilization with 0.005% saponin (diluted in high-K+ Tyrode solution devoid of Ca^{2+}: 140 mmol/L KCl, 1 mmol/L EGTA, 1 mmol/L MgCl, 10 mmol/L HEPES; pH 7.4). After permeabilization, cardiomyocytes were equilibrated with internal solution (9 mmol/L NaCl, 0.5 mmol Na^+-pyruvate and 0.5 mmol/L maleic acid, Na^+ salt – for a total of 10 mmol/L Na^+ concentration - 20 mmol/L KCl, 100 mmol/L K^+-gluconate, 15 mmol/L HEPES, 5 mmol/L EGTA, 5 mmol/L MgATP, 0.5 mmol/L K^+-phosphate monobasic, 0.25 mmol/L K^+-ADP dehydrate, 1 mmol/L free [Mg^{2+}]; pH 7.2) containing 100 nmol/L free [Ca^{2+}] to measure mitochondrial Ca^{2+} load at conditions which mimic basal cytosolic [Ca^{2+}], and Rhod-2 fluorescence quantified at steady-state. To measure mitochondrial Ca^{2+} efflux, the decline in Rhod-2 fluorescence was recorded after cardiomyocytes were exposed to the same internal solution as above but devoid of Ca^{2+}. Calibration of mitochondrial Ca^{2+} was done at the end of each protocol, with F_{min} and F_{max} determined at 0 and 200 nmol/L [Ca^{2+}], respectively, in the internal solution containing 5 μmol/L ionomycin. For

all internal solutions, $[Ca^{2+}]$ and $[Mg^{2+}]$ were adjusted appropriately using CaBuf software (G. Droogmans, Leuven, Belgium). Experiments were performed with confocal microscopy (Leica LCS Live system) using 514 nm excitation and 580-620 emission. Images were analysed using a custom-made macro in ImageJ.

$[Ca^{2+}]_i$ transient measurements and spontaneous Ca^{2+} release event assessment. Intracellular Ca^{2+} ($[Ca^{2+}]_i$) transients (Fura-2AM) and unloaded cell shortening were simultaneously measured under 3Hz field-stimulation at 35°C in standard 1.4 mM Ca^{2+} Tyrode buffer with or without selected treatments. Cardiomyocytes were paced until steady-state was reached, at which point pacing was paused for 1 minute to assess the probability of spontaneous $[Ca^{2+}]_i$ release. Following the pause, 3 Hz pacing was resumed and steady-state reached again before rapid application of caffeine (10 mmol/L) to determine total SR Ca^{2+} load. The amplitude of the $[Ca^{2+}]_i$ transient under steady-state and during caffeine (SR content) was calculated as the difference between diastolic and systolic Ca^{2+} fluorescence. Any cell which did not show a rapid caffeine response was excluded from the analysis. The rate of the steady-state $[Ca^{2+}]_i$ transient decay was best fit by a double exponential (Clampfit10.5, Axon Instruments) and tau_1 was used for comparisons between groups. Calculations for the rates of Ca^{2+} removal – represented as K_{SR} and K_{NCX} – were carried out as previously described[56]. Briefly, the $[Ca^{2+}]_i$ transient decay upon caffeine application was attributed to Sodium/Calcium Exchanger (NCX) transport (K_{NCX}) while the decay of the steady-state $[Ca^{2+}]_i$ transient was representative of sum of the SR Ca^{2+}-ATPase (SERCA) and NCX transport (where the difference indicates K_{SR}).

Patch clamping. Whole-cell voltage experiments were carried out in mouse LV cardiomyocytes at 36°C with 3Hz pacing using an Axopatch 200B amplifier and Digidata 1322A data-acquisition system (Axon Instruments). Recordings of the L-type Ca^{2+} current ($I_{Ca,L}$) were measured during a 200ms test pulse from a holding potential of -40 mV to 0mV in the rupture patch mode. After 3 min of equilibration, membrane rupture was allowed before using cells for further experiment. IV curves were recorded using a stepdepolarization protocol (test potentials in the range -40 to +60 mV in 5 mV increments from a holding potential of -40 mV) and $I_{Ca,L}$ was measured as the difference between the current measured at the end of 200 ms test pulse and the peak inward current. Activation curves were calculated from IV curves. After IV recordings, cells were stimulated at 3Hz for 1 min before continuing with (1) steady-state inactivation protocol or (2) rapid caffeine (10 mmol/L) application to measure the caffeine-induced NCX current (I_{NCX}). For steady-state inactivation, $I_{Ca,L}$ at the test step of 0 mV was normalized to the maximum current and plotted against the potential of a 1s-long inactivating pre-pulse between –60 and +130 mV. A Boltzmann function was used to fit the normalized values. At the end of each recording, a bath solution containing 300 µmol/L cadmium was applied to block Cd-sensitive $I_{Ca,L}$. To correct for differences in myocyte size, currents are expressed as current densities (pA/pF). Solutions used for recordings: *bath solution* (in mM) – NaCl -140; KCl-6, $MgCl_2$ – 1, HEPES – 10, Glucose - 10, $CaCl_2$ - 1.4; *pipette solution* (in mM) – HEPES - 10, K-glutamate - 120, KCl -130, K-ATP - 5, $MgCl_2$ - 5, Na_2-creatine phosphate - 3.6, NaCl - 2.8, cAMP – 0.05.

Assessment of $[Ca^{2+}]_i$ dynamics and RyR leak**.** The protocol was performed as previously described[29] in Fura-2AM loaded myocytes, where leak was measured as the difference of diastolic $[Ca^{2+}]_i$ under 0Na/0Ca^{2+} conditions in the absence or presence of tetracaine (1 mmol/L). RyR leak was expressed as a proportion of the

total SR Ca^{2+} load. The rate of Ca^{2+} transient oscillations occurring during tetracaine or upon washout was expressed as the percentage of myocytes that developed oscillations for each isolation.

Arrhythmia susceptibility in perfused, beating hearts. Experiments were conducted at the University of Birmingham, UK according to the Animals (Scientific Procedures) Act 1986 and approved by the Home Office (Home Office References 30/2967 and the local institutional review board. Deep terminal anaesthesia was achieved by inhalation of 5% isoflurane with an O_2 flow rate of 3 L/min. Murine hearts were rapidly extracted under heparinization, the aorta was cannulated, retrogradely perfused and mounted on a vertical Langendorff-apparatus (Hugo Sachs/Harvard Apparatus, Germany) for coronary perfusion with Krebs-Henseleit (KH) buffer aiming for constant aortic pressure (100 ± 5 mmHg), coronary flow rate (4 ± 1ml/min) and temperature (36 ± 1 °C). After 3 min of perfusion, stimulation was conducted via an octapolar EP-catheter inserted through a small hole in the right atrium into the right ventricle. Atrioventricular block was induced mechanically by applying localised pressure to the septum just under the tricuspid valve with a small set of tweezers. The right ventricular septum was burst-paced using two electrodes of the octapolar EP-catheter at different pacing cycle lengths (PCLs) between 60 and 200 ms. Spontaneous arrhythmias and induced arrhythmias during burst pacing were analysed and graded by severity.

Ex vivo I/R. Mice were heparinized (300U) and anaesthetized with ketamine (75mg/kg) plus medetomidine hydrochloride (1mg/kg), with the adequacy of anaesthesia confirmed by the absence of a pedal reflex. Hearts were quickly excised and immersed in KH buffer. The aorta was then cannulated onto the Langendorff perfusion system for retrograde perfusion. The heart was perfused with 37°C KH buffer, gassed with 95% O2/ 5% CO2, at 2mL/min and cardiac function was assessed using a fluid-filled balloon inserted into the left ventricle, which connected to a pressure transducer and a PowerLab system (ADInstruments). Left ventricular developed pressure (LVDP), calculated by the difference between systolic and diastolic pressure, was recorded continuously via LabChart software v.7.0. After 25 mins equilibration, hearts were subjected to 25 mins global ischemia followed by 60 mins of reperfusion. In a sub-group of experiment, 10 µmol/L Ned-19 was added in the KH buffer at the time of reperfusion. Only male mice were used to assess infarct size analysis, whereas an equal mix of male and female mice were used for functional measurements.

Triphenyltetrazolium chloride (TTC) staining. Following *ex vivo* I/R, hearts were removed from the Langendorff, briefly frozen and then sliced into 6, 1 mm thick transverse sections. In order to distinguish viable (stained) vs necrotic (pale, unstained) tissue, sections were incubated in 1% TTC for 30 mins at 37°C. Sections were then scanned and the area of infarction (TTC negative) quantified as a percentage of the area at risk (entire area of the section) using ImageJ.

Statistics. All experimentation and data analysis, apart from immunoblots, were conducted blinded to genotype. Data were checked for normality of distribution prior to statistical analysis using a Shapiro-Wilk test. Comparisons between normally distributed data were performed using either a Student’s t-test or an analysis of variance (ANOVA) with Bonferroni’s correction, as indicated in the figures. Non-normally distributed data were compared using the Mann-Whitney test or Kruskal-Wallis test. For baseline Ca^{2+} handling data in cardiomyocytes, analyses were carried

out in RStudio using a hierarchical statistical method[20], which takes in to consideration clustering of single cells per animal and corrects for that in the statistical analysis. This method has been shown to be better suited to eliminating false-positives as a result of using individual cells as a single ‘n’. Results were considered statistically significant at a p-value <0.05.

Supplemental Tables:

Supplemental Table I: Echocardiographic evaluation in WT and KI mice.

	WT (n=7)	KI (n=7)	P value
LV mass (mg/BW)	2.50 ± 0.31	2.52 ± 0.18	0.94
LVIDs (mm)	2.27 ± 0.24	2.32 ± 0.27	0.88
LVIDd (mm)	3.33 ± 0.15	3.15 ± 0.22	0.50
LVPWs (mm)	1.08 ± 0.06	1.07 ± 0.09	0.33
LVPWd (mm)	0.65 ± 0.03	0.71 ± 0.07	0.52
EF (%)	60 ± 8	54 ± 7	0.55
FS (%)	32 ± 5	28 ± 4	0.47
HR	447 ± 28	497 ± 16	0.15

Data are given as Mean ± SEM; statistical comparisons were done using a Student's t-test. LV mass: left ventricular mass normalized to body weight (BW); LVIDs,d: left ventricular internal diameter at the end of systole (s) and at the end of diastole (d); LVPWs,d: left ventricular posterior wall size at the end of systole (s) and at the end of diastole (d); EF: ejection fraction; FS: fractional shortening; HR: heart rate in beats per minute.

Supplemental Table II: Percentage of PKARIα disulfide formation under each experimental condition.

Condition	% PKARIα disulfide formation	Experiments Used
Single Cells		
WT adult mouse LV cardiomyocytes - freshly isolated (n=5)	27.6 ± 12.0%	Cardiomyocyte area
WT adult mouse LV cardiomyocytes - 4 hrs culture (n=5)	75.6 ± 6.4%**	Confocal & STED imaging
WT adult mouse LV cardiomyocytes – 24 hrs culture (n=5)	71.5 ± 10.5%**	FRET imaging
WT adult mouse LV cardiomyocytes – H_2O_2 treatment (n=11)	83.0 ± 6.9%**	FRET imaging
WT adult mouse LV cardiomyocytes – fura2 loaded (paced and unpaced; n=5)	96.7 ± 7.1%**	Ca^{2+} dynamics & patch clampling
prkar-/- mouse embryonic fibroblasts – 48 hrs culture (n=3)	59.8 ± 7.8%	FRAP imaging
Neonatal rat cardiomyocytes (n=3)	Not assessed	STED imaging (AKAP peptide disruption)
Tissue Homogenates		
Human atrial biopsies (n=18)	pre I/R – 15.2% (12.3-25.6)<br>post I/R – 31.7% (12.9-44.0)$^{\#}$	Biochemistry
Adult mouse LV – transient LAD occlusion (n=5, sham; n=4, I/R and remote)	35.6 ± 6.4%- sham<br>71.2 ± 1.4% – remote†<br>84.9 ± 6.5% – I/R$^{\dagger\ \ddagger}$	Biochemistry
Adult mouse LV – baseline, rapidly processed (n=6)	30.6 ± 13.8%	Phenotypic evaluation (qPCR & Western Blotting)

Data are given as Mean ± SD or Median with IQR, where appropriate. Percentages calculated from non-reduced immunoblotting of PKARIα under each condition. LV: left ventricle; STED: stimulation emission depletion; FRET: fluorescence resonance energy transfer; FRAP: fluorescence recovery after photobleaching; WT: wild-type; LAD: left anterior descending artery; I/R: ischemia-reperfusion. **p<0.0001 vs freshly isolated cardiomyocytes; one-way ANOVA with Bonferroni's corrections. $^{\#}$p<0.05 vs pre I/R from same patient; paired Student's t-test. †p<0.001 vs Sham, ‡p<0.05 vs Remote; one-way ANOVA with Bonferroni's correction.

Supplemental Table III: Baseline LV parameters in Langendorff-perfused WT and KI hearts.

	WT (n=9)	KI (n=8)	WT + Ned-19 (n=7)	KI + Ned-19 (n=7)	P value
LVDP (mmHg)	87.3 ± 3.4	92.5 ± 6.3	103.8 ± 5.2	93.7 ± 3.9	0.60
Heart Rate (beats/min)	286.5 ± 21.5	292.9 ± 20.1	293.1 ± 5.5	293.2 ± 20.2	0.30
CPP (mmHg)	97.8 ± 4.9	89.6 ± 7.5	112.6 ± 7.4	104.8 ± 12.8	0.25

Data are given as Mean ± SEM and statistical comparisons were done using a two-way ANOVA. LVDP, left ventricular developed pressure; CPP, coronary perfusion pressure.

Supplemental Figures:

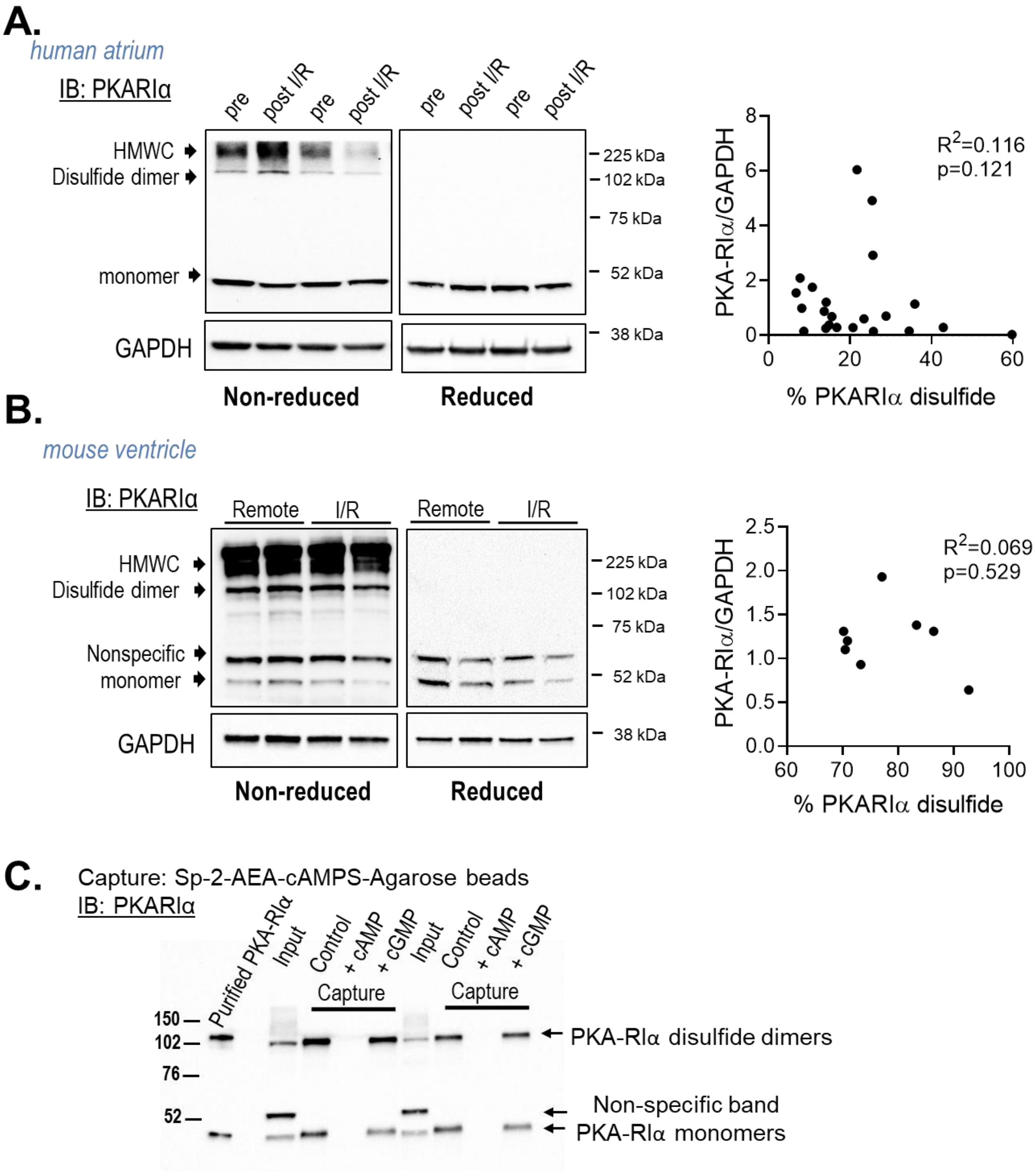

Supplemental Figure I: (**A**) Chemical reduction (10% 2-mercaptoethanol) of PKARIα disulfide dimers and high molecular weight complexes (HMWC) to a single monomeric PKARIα band in human atrial biopsies (*pre*, prior to cardiopulmonary bypass and *post I/R*, after cardioplegia and reperfusion). There was no correlation between PKARIα disulfide formation (as quantified in Figure 1B under non-reduced conditions) and total expression of PKARIα protein (quantified under reduced conditions and normalized to GAPDH), indicating that the PKARIα oxidation state does not lead to protein degradation. Linear regression was run on the log-transformed data to achieve normal distribution for statistical analysis, n=22 (**B**) Similarly, in mouse LV tissue from the remote and I/R region, 10% 2-mercaptoethanol treatment resulted in complete reduction of PKARIα disulfide dimers and HMWC to a monomeric PKARIα band, with no correlation observed between the extent of PKARIα disulfide formation (as quantified in Figure 1D under non-reduced conditions) and total PKARIα protein

(quantified under reduced conditions and normalized to GAPDH); Linear regression, n=8 (**C**) For appropriate identification of PKARIα monomers and disulfide dimers in immunoblots from mouse LV tissue, cAMP-affinity capture, which selectively enriches for all cAMP-interacting proteins was used. Purified human PKARIα protein was used as a positive control for molecular weight identification of PKARIα monomers and disulfide dimers. Input samples from mouse LV homogenates showed the appearance of 3 immuno-reactive bands – two at molecular weights corresponding to the purified protein monomer (approx. 52 kDa) and disulfide dimer (approx. 105 kDa), and a third band appearing around 60 kDa. Following cAMP-affinity capture, however, the band at 60 kDa was no longer detected, indicating that this is a non-specific reaction of the PKARIα antibody. To ensure specificity of the affinity capture, an aliquot of each samples was pre-incubated with cAMP (60 μmol/L), which saturates cAMP-binding sites to prevent binding, or cGMP (60 μmol/L) as a negative control.

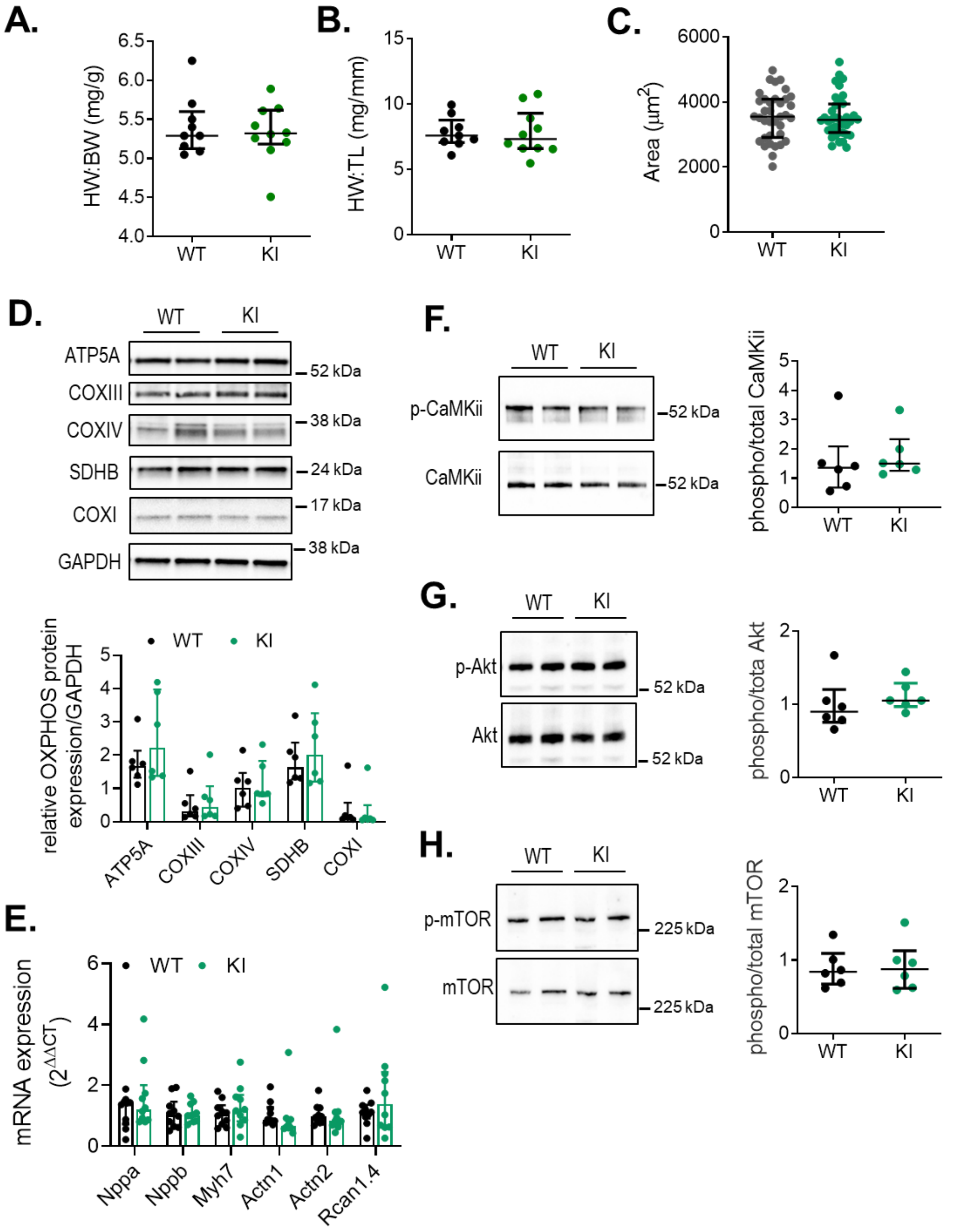

Supplemental Figure II: Phenotypic evaluation of cardiac structure, function and signaling between WT and KI hearts at baseline. Compared to WT, KI mice showed no difference in cardiac morphology, including similar (**A**) heart weight-to-body weight (HW:BW) and (**B**) heart weight-to-tibia length (HW:TL) ratios (n=9 for WT and n=10 for KI)*;* (**C**) comparable LV cardiomyocyte area (length x width; 12 cells per mouse measured, n=3 mice per genotype); (**D**) equal levels of LV mitochondrial content (assessed by OXPHOS protein content, normalized to GAPDH, n=6/grp); (**E**) similar expression of hypertrophic genes in LV tissue (n=10/grp, normalized to the

housekeeper PPIA). There was also no genotype-dependent differences found in neurohumoral signaling pathways, including: (**F**) CaMKii δ auto-phosphorylation (Thr^{247}); (**G**) phospho-dependent activation of Akt (Ser^{473}); and (**H**) phospho-dependent activation of mTOR (Ser^{2448}) For F-H, n=6. All data are shown as median with IQR. Pairwise comparison between genotypes was made using a Mann-Whitney non-parametric test.

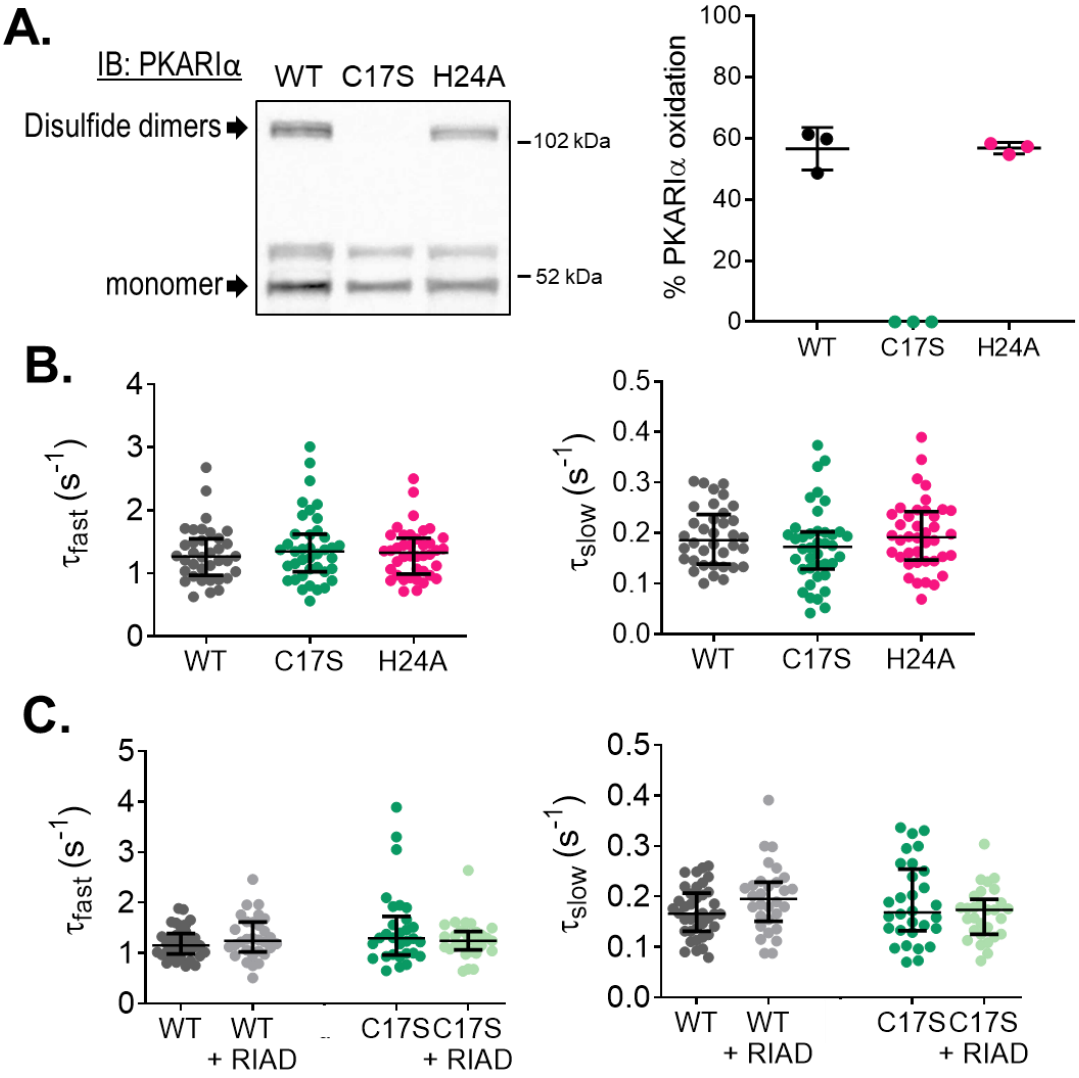

Supplemental Figure III: (**A**) Representative non-reducing immunoblot from *prkar1a*$^{-/-}$ MEF cells expressing WT (grey), C17S (green) or H24A (pink) forms of GFP-tagged PKARIα. Quantified percentage of PKARIα disulfide dimers and monomers demonstrate that the WT and H24A mutant form of PKARIα -GFP contain disulfide bonds under basal conditions (59.8 ± 7.8% and 58.4 ± 4.3%, respectively), whereas the C17S mutant form does not. Data shown with mean ± SD; n=3. (**B and C**) Fluorescence recovery after photobleaching recovery curves were best-fit using a bi-exponential equation to obtain diffusion time-constants (tau, τ). The fast component (*left*) represents expressed GFP-tagged RIα in the monomeric form while the slow component (*right*) represents diffusion of RIα within the PKA holoenzyme structure. (**B**) Diffusion time constants for cells expressing the WT (grey), C17S (green) or H24A (pink) form of GFP-tagged PKARIα. (**C**) Diffusion time constants for WT- or C17S-expressing cells co-transfected with either RIAD or GFP control. All data points are shown with median and IQR indicated. Statistical testing was done on log-transformed data (to achieve normal data distribution) using a one-way ANOVA; n = 30-39 cells per group, from 3 independent passages.

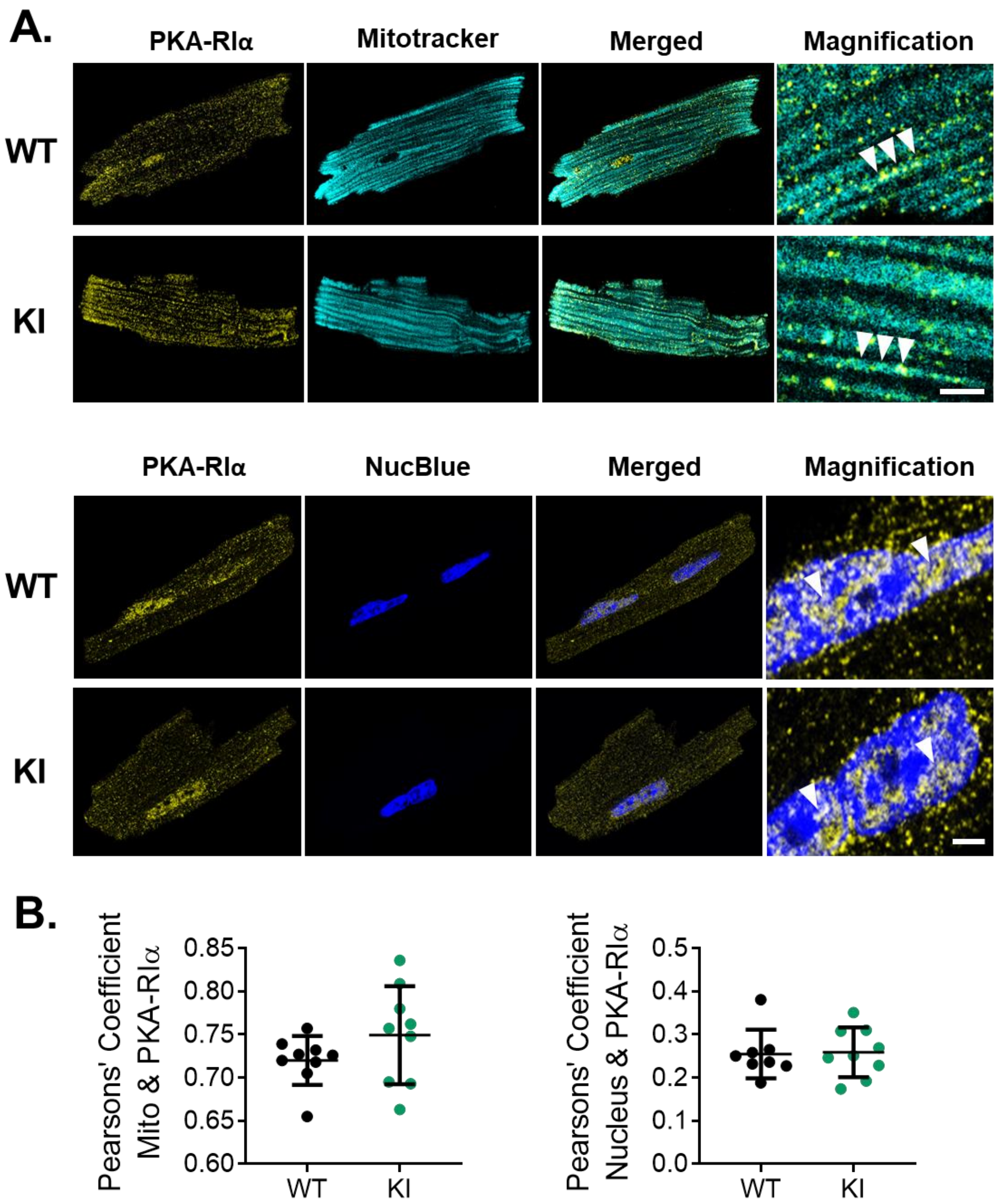

Supplemental Figure IV: Confocal imaging showed similar degrees of PKARIα co-localization with the mitochondria (Mitotracker, *top*) and nucleus (NucBlue, *bottom*) in WT and KI LV cardiomyocytes, observed both (**A**) visually, with arrows highlighting regions of co-localization, and (**B**) quantitatively using measurement of the Pearsons' correlation coefficient. Data shown as mean ± SD; n= 9 cardiomyocytes per group, each from 3 mice. Scale bars = 2μm

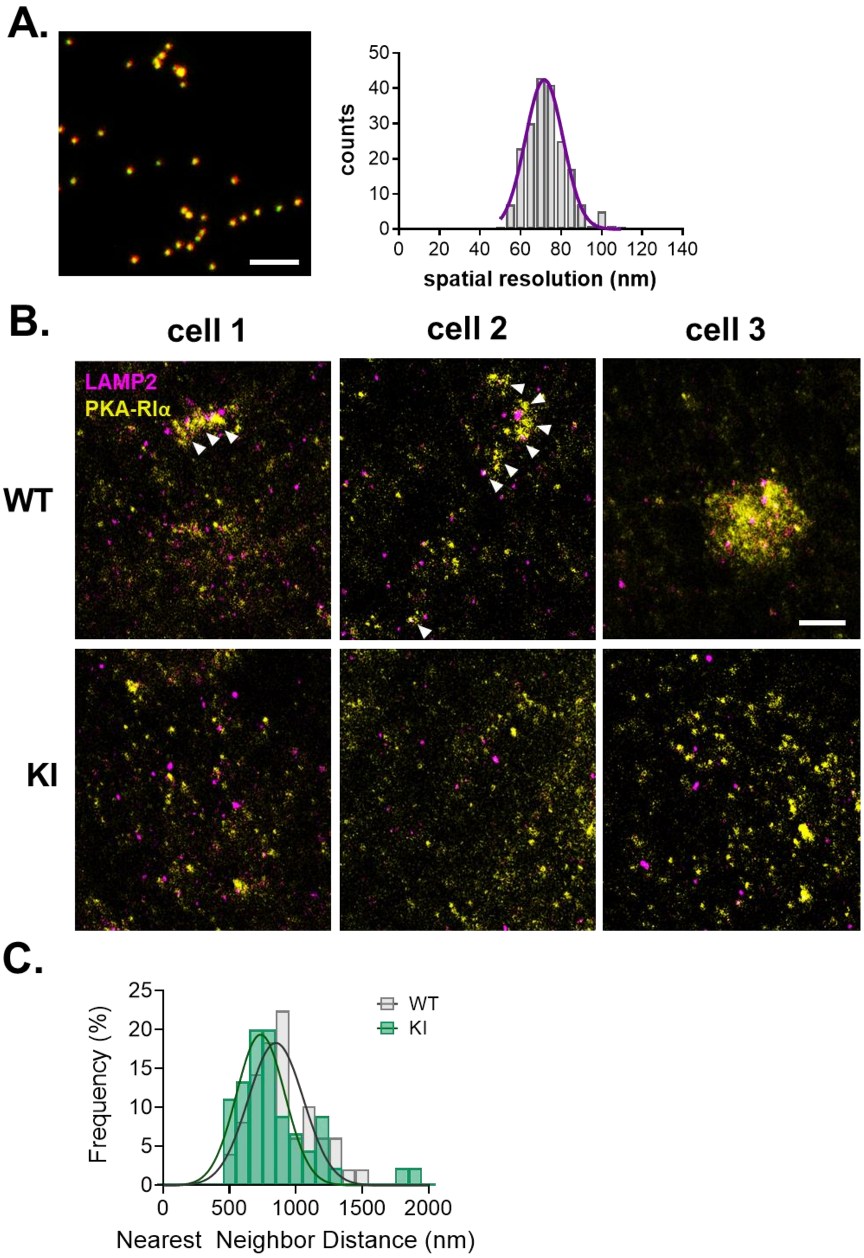

Supplemental Figure V. (**A**) Beads (50nm) co-labelled with AF488 and AF594 were used to determine the spatial resolution of STED imaging under our experimental conditions. The spatial resolution, quantified as the full width at half maximum fluorescence intensity (FWHM) in the 488 wavelength, was 72.9 ± 0.7nm. 200 individual beads, imaged from 5 separate sections, were used to calculate the mean FWHM. (**B**) Additional STED images showing PKARIα clustering near LAMP-2 positive lysosomes in WT cardiomyocytes (*indicated by arrows*), but not KI cardiomyocytes. Scale bars = 1 µm. (**C**) The spatial distribution of lysosomes (determined using STED by measuring the closeness of each lysosome to its nearest lysosomal neighbour) was not different between WT and KI cardiomyocytes. Differences between genotypes were assessed using the Kolmogorov–Smirnov test on cumulative distributions; n=45-49/N=6 per genotype.

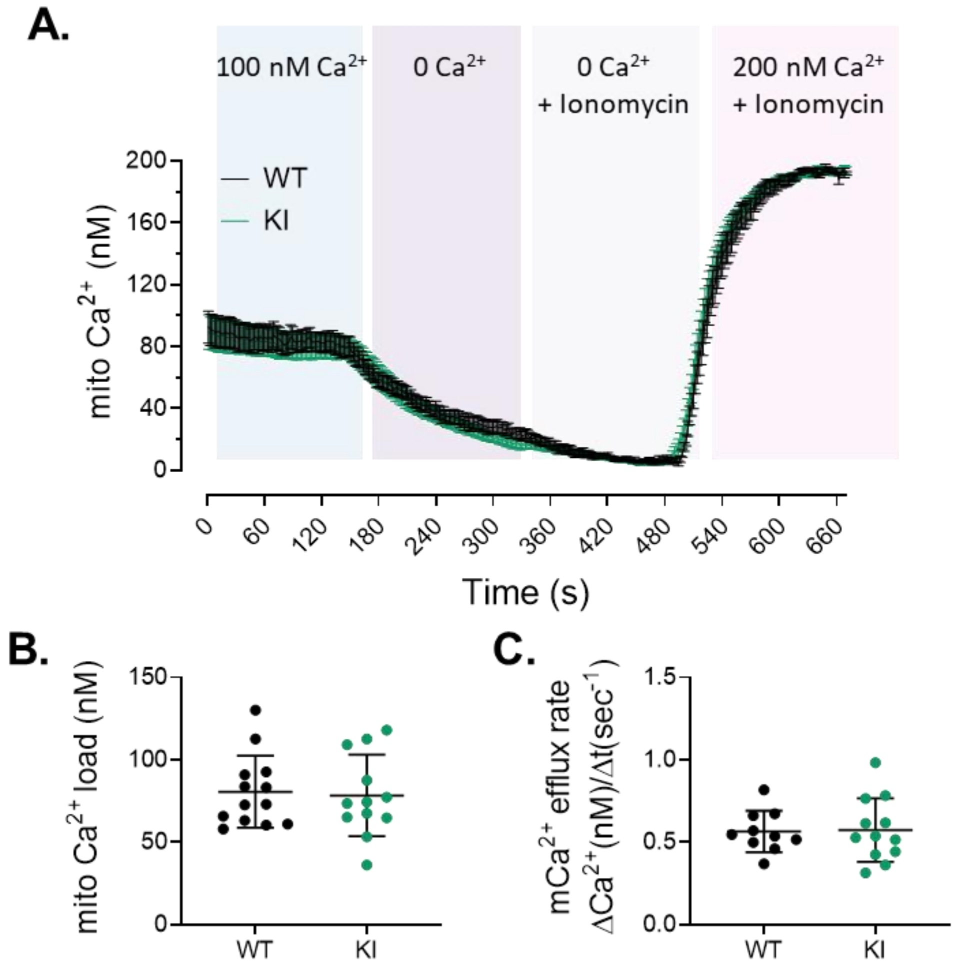

Supplemental Figure VI. Basal mitochondrial calcium handling is similar in WT and KI cardiomyocytes. (**A**) Dynamic measurement of calibrated Rhod-2 fluorescence in permeabilized cardiomyocytes from WT and KI mice allowed for the quantification of mitochondrial Ca^{2+} load (following perfusion with internal solution containing 100 nmol/L Ca^{2+}) and efflux rates (after rapid switching to internal solution devoid of Ca^{2+}). Solutions containing 0 or 200 nmol/L Ca^{2+} with 5 μmol/L Ionomycin were used at the end of each protocol to calibrate Ca^{2+} concentrations for each cardiomyocyte. Traces shown are the mean ± SEM of all traces acquired for each genotype. (**B**) Quantification of steady-state mitochondrial Ca^{2+} load and (**C**) the rate of Ca^{2+} efflux show no difference between genotypes. All data points are shown with mean ± SD indicated; n=12-13 myocytes from N=3 mice per genotype.

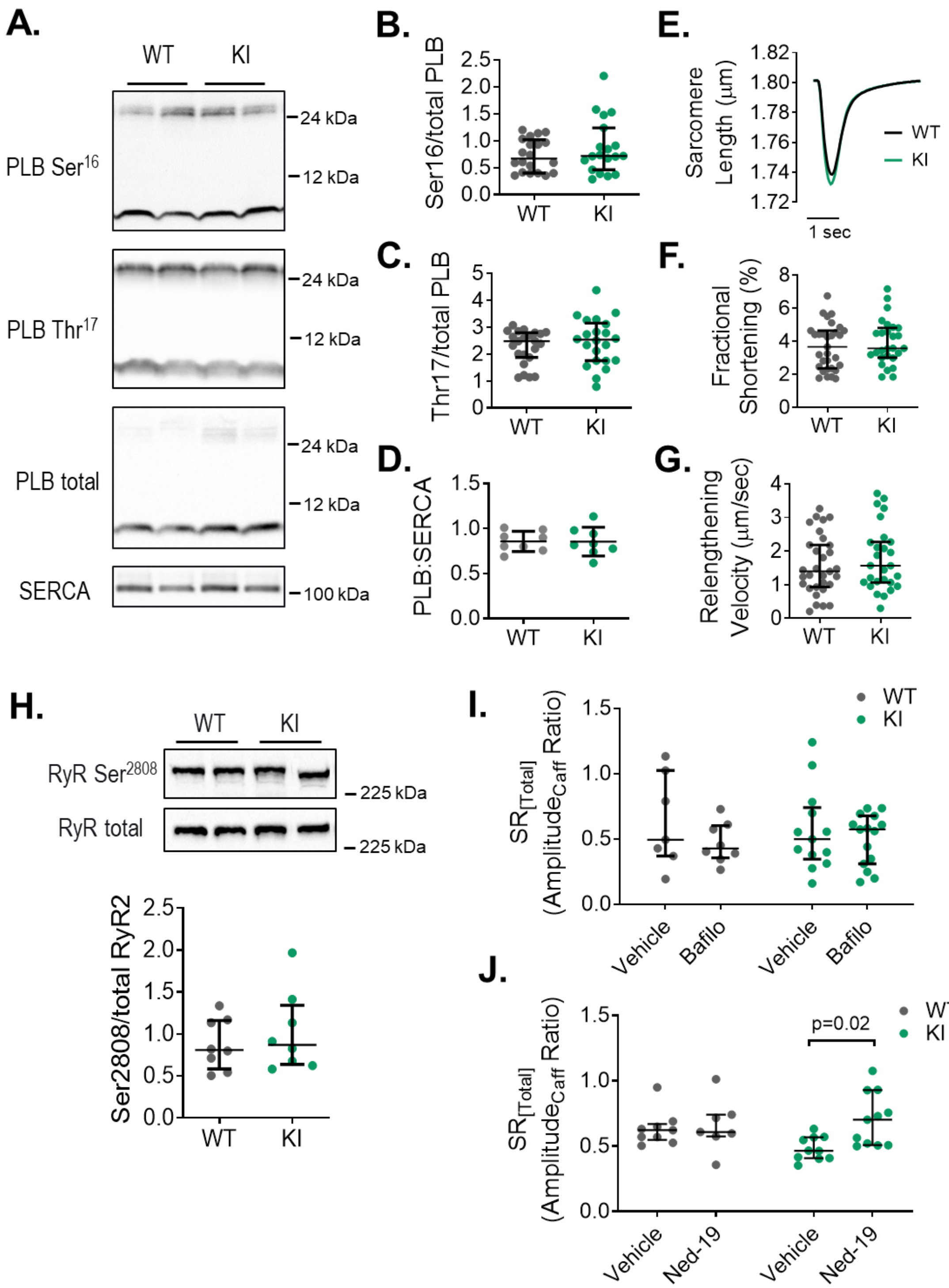

Supplemental Figure VII: (A) Representative immunoblots of phospho-Ser16, phospho-Thr17 and total PLB in WT and KI LV homogenates. Phosphorylated PLB levels were assessed under non-reducing conditions to preserve the pentameric state (MW approx. 25 kDa). Total PLB levels were assessed under reduced conditions after treatment with λ-phosphatase (400U/μL) to circumvent the problem of phosphorylation-dependent differences in antibody binding affinity known to occur with most commercially available total PLB antibodies (including the mAb A1 clone used here – A010-14; Badrilla). Quantitation of PLB phosphorylation at (**B**) Ser16 and (**C**) Thr17, as well as (**D**) the PLB-to-SERCA ratio showed no difference between

genotypes. All data points shown with the median and IQR. Statistical testing was done using a Mann-Whitney nonparametric test; n = 19 hearts per genotype for B and C, n=8 per genotype for D. (**E**) Dynamic changes in sarcomere length (electrically stimulated at 3 Hz, 35±1°C) were measured in a subset of WT and KI cardiomyocytes simultaneous to the Ca^{2+} transient measurements shown in Figure 6. Neither the (**F**) fractional shortening, presented as a percentage of diastolic sarcomere length, or (**G**) the relengthening velocity were found to be different between genotypes. All data points are shown with the median and IQR; statistical analysis was done using a hierarchical model on normally-distributed data, with logarithmic transformation applied prior to statistical testing to achieve a normal distribution; n = 27-31 cardiomyocytes from 8 mice per genotype. (**H**) Representative immunoblot and quantification of RyR phosphorylation at the known PKA site, Ser2808. Mann-Whitney nonparametric test; n = 8 animals per genotype. (**I** and **J**) Quantification of SR Ca^{2+} content, using rapid caffeine application (10 mmol/L), in Fura-2 loaded cardiomyocytes isolated from WT or KI mice following 10-15 mins treatment with either (**I**) bafilomycin A1 (Bafilo; 100 nmol/L) or (**J**) Ned-19 (5 µmol/L). Equivalent volumes of DMSO were used as vehicle controls. Two-way ANOVA with Bonferonni's correction. n=7-11 cardiomyocytes from N=3 mice/genotype for Bafilo and n=7-11 cardiomyocytes from N=3 mice/genotype for Ned-19.

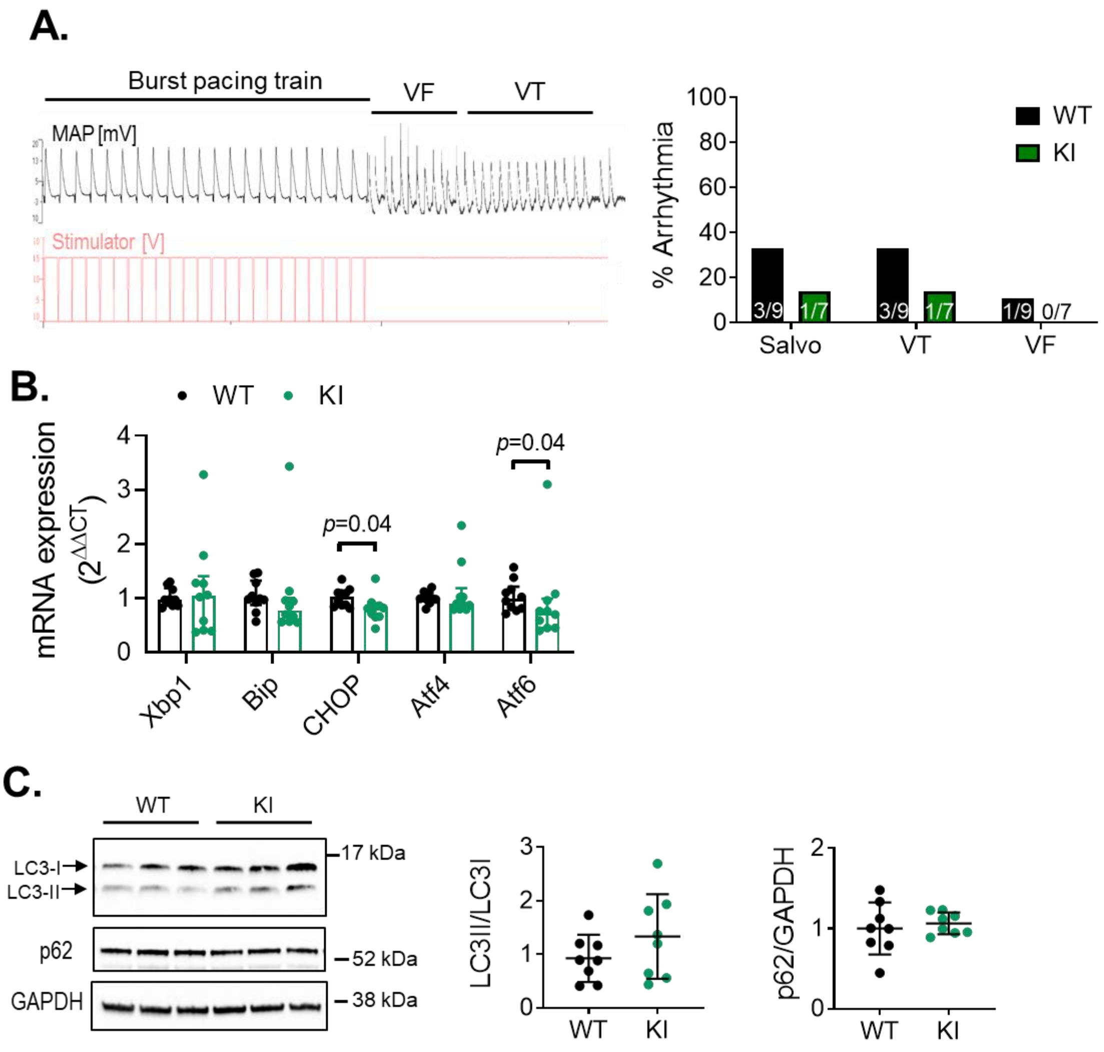

Supplemental Figure VIII: (**A**) Assessment of burst-pacing induced arrhythmias was done in isolated, perfused hearts following AV-node crush, which allowed for isolation of arrhythmias which were ventricular in origin. The number of hearts which developed episodes of salvo, ventricular tachycardia (VT; >5beats) and ventricular fibrillation (VF) was determined blinded to genotype, with comparisons made using a Fisher's exact test on the absolute number of hearts. No significant differences were found between genotypes for any forms of arrhythmia assessed; n-numbers as indicated. (**B**) Expression level of genes in LV tissues which are regulated by all three branches of the ER unfolded protein response pathways. n=6/genotype, normalized to the housekeeper gene PPIA. All data points shown with the median and IQR. Mann-Whitney test for each pairwise comparison. (**C**) *Left*, immunoblot assessment of the conversion of LC3-I to LC3-II and p62 degradation as markers of autophagosome-lysosome pathway (ALP) activation. *Right*, quantification of each ALP marker in LV homogenates from WT and KI mice. n=8/genotype. Mean ± SD, Student's t-test.

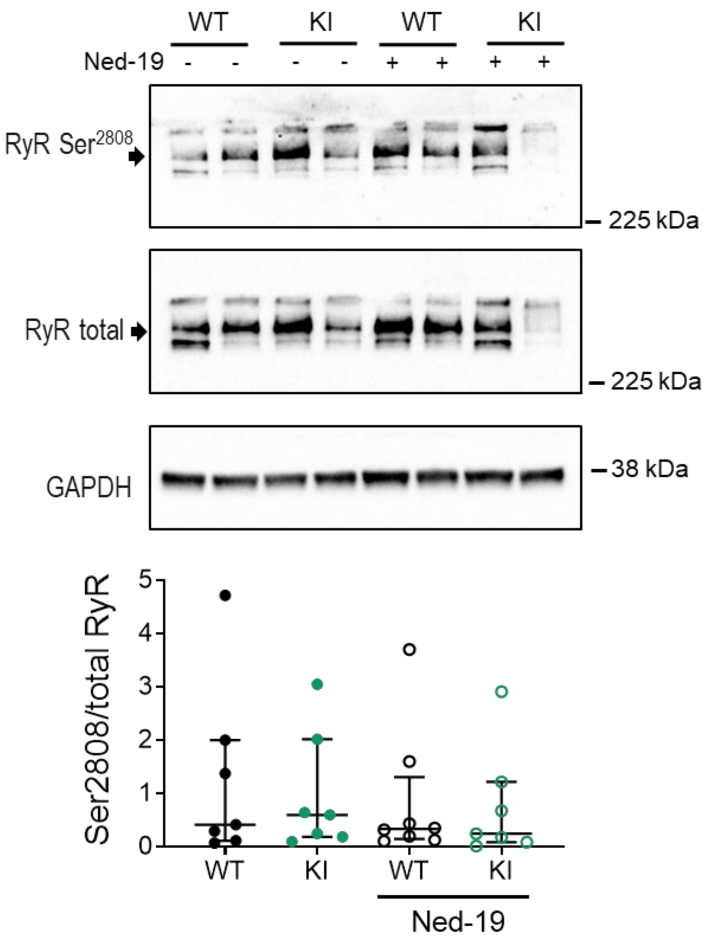

Supplemental Figure IX: No difference in RyR phosphorylation at the PKA-site (Ser2808) was found between WT and KI hearts following I/R. Median and IQR, n=7/group; two-way ANOVA performed on log-transformed data to achieve normal distributions.
